# An Easily Expandable Multi-Drug LC-MS Assay for the Simultaneous Quantification of 57 Oral Antitumor Drugs in Human Plasma

**DOI:** 10.3390/cancers13246329

**Published:** 2021-12-16

**Authors:** Niklas Kehl, Katja Schlichtig, Pauline Dürr, Laura Bellut, Frank Dörje, Rainer Fietkau, Marianne Pavel, Andreas Mackensen, Bernd Wullich, Renke Maas, Martin F. Fromm, Arne Gessner, R. Verena Taudte

**Affiliations:** 1 Institute of Experimental and Clinical Pharmacology and Toxicology, Friedrich-Alexander-Universität Erlangen-Nürnberg (FAU), 91054 Erlangen, Germany; niklas.kehl@fau.de (N.K.); katja.schlichtig@fau.de (K.S.); pauline.duerr@uk-erlangen.de (P.D.); renke.maas@fau.de (R.M.); martin.fromm@fau.de (M.F.F.); 2 Comprehensive Cancer Center Erlangen-EMN (CCC ER-EMN), University Hospital Erlangen, 91054 Erlangen, Germany; laura.bellut@uk-erlangen.de (L.B.); frank.doerje@uk-erlangen.de (F.D.); rainer.fietkau@uk-erlangen.de (R.F.); marianne.pavel@uk-erlangen.de (M.P.); andreas.mackensen@uk-erlangen.de (A.M.); bernd.wullich@uk-erlangen.de (B.W.); 3 Pharmacy Department, University Hospital Erlangen, 91054 Erlangen, Germany; 4 Department of Urology, University Hospital Erlangen, 91054 Erlangen, Germany; 5 Department of Radiation Oncology, University Hospital Erlangen, 91054 Erlangen, Germany; 6 Department of Internal Medicine 1, University Hospital Erlangen, 91054 Erlangen, Germany; 7 Department of Internal Medicine 5, Haematology and Oncology, University Hospital Erlangen, 91054 Erlangen, Germany

**Keywords:** oral antitumor drug, Orbitrap mass spectrometer, exposure measurement, liquid chromatography mass spectrometry, kinase inhibitors

## Abstract

**Simple Summary:**

Oral antitumor therapy has significantly improved clinical outcomes in multiple tumor entities. However, following a standard dosing regime, strong interindividual variability in patients’ plasma concentrations can be observed for many oral antitumor drugs. This results in risks of reduced therapeutic effect and increased side effects. Monitoring these variable plasma concentrations is an important tool in evaluating multiple factors influencing drug exposure and, if necessary, adjusting therapeutic doses. Here, we developed a method for the simultaneous measurement of 57 oral antitumor drug plasma concentrations. Detection and quantification were achieved using liquid chromatography coupled to an Orbitrap mass spectrometer, which can be easily expanded to newly approved oral antitumor drugs in the future. Applicability of the method was proven by measuring 71 plasma samples from 39 patients undergoing oral antitumor therapy. In summary, the developed method provides an important tool for exposure measurements of oral antitumor drugs.

**Abstract:**

Oral anticancer drugs have led to significant improvements in the treatment of multiple tumor entities. However, in patients undergoing oral antitumor therapy, plasma concentrations are highly variable, resulting in risks of reduced therapeutic effects or an increase in side effects. One important tool to reduce this variability is therapeutic drug monitoring. In this work we describe a method to simultaneously quantify the plasma concentrations of 57 oral antitumor agents. Quantification of these drugs was achieved using liquid chromatography coupled to an Orbitrap mass spectrometer. The method was fully validated according to the FDA guidelines and constitutes a simple and robust way for exposure monitoring of a wide variety of oral anticancer drugs. Applicability to clinical routine was demonstrated by the analysis of 71 plasma samples taken from 39 patients. In summary, this new multi-drug method allows simultaneous quantification of 57 oral antitumor drugs, which can be applied to exposure monitoring in clinical studies, taking into account the broad variety of oral antitumor drugs prescribed in clinical routine.

## 1. Introduction

Oral anticancer drugs play an important role in the treatment of multiple tumor entities [1] and have contributed to significantly improved treatment outcomes for cancer patients [2,3]. During the last two decades, a wealth of oral anticancer drugs has been approved worldwide [4,5]. For instance, 76 oral anticancer drugs were approved in Germany during this period, whereby approximately 40 of them are protein kinase inhibitors [6]. This number is expected to increase in the upcoming years, as there are many drugs in preclinical and clinical development [7]. Despite major clinical improvements conferred by oral anticancer drugs, there are still issues with the potential for optimization of clinical outcome, therapeutic safety, and patient satisfaction [8].

Most of the oral anticancer drugs—especially protein kinase inhibitors—are prescribed in a one dose fits all approach [6,9,10]. However, several clinical studies demonstrated considerable interindividual differences in drug exposure despite identical dosing [5,9,10,11,12,13,14], which can be considered as suboptimal. Imatinib, for example, showed varying plasma concentrations by a factor of 60 [15]. Many aspects can contribute to the described high interindividual variability. For instance, patient adherence differs greatly with reported values between 46 and 100% [6,16]. Furthermore, approximately 50% of oral anticancer drugs showed variable absorption rates when taken with or without food [6,17,18]. The co-administration of acid-reducing agents (e.g., proton-pump inhibitors) can diminish absorption of multiple oral anti-cancer drugs [6,19]. Besides, a large proportion of these drugs are predominantly metabolized via CYP3A4 [6,20]. Concurrent administration of CYP3A4 inhibitors (e.g., clarithromycin), nutrition (e.g., grapefruit-products), or herbal medicines (e.g., St. John’s Wort) can alter pharmacokinetics of oral anticancer drugs in a clinically relevant manner [21]. An analysis of medication errors in 202 patients treated with oral anticancer drugs illustrated that 47% of all detected medication errors involving these drugs had the potential to affect oral anticancer drug-pharmacokinetics [22]. Approximately half of these errors were caused by the patients [22].

Some studies demonstrated an association between low plasma concentrations and poorer treatment outcomes for a number of oral anticancer drugs such as alectinib, imatinib, and vemurafenib [5,9]. Similarly, patients with high drug plasma concentrations are more likely to develop side effects in comparison to patients with lower exposures [9,23,24]. In clinical routine, multiple aspects have the potential for optimization of both medication safety and clinical outcome. In a recently published randomized controlled trial we reported a significant reduction of severe side effects, medication errors, and treatment discontinuations as well as improved adherence in patients treated with oral anticancer drugs through intensified clinical pharmacological/pharmaceutical care [8].

A crucial measure to evaluate and minimize the above mentioned factors is the determination of a patient’s plasma concentration via therapeutic drug monitoring (TDM) [5,14,25]. Considering the correlation of drug exposure with therapeutic success, TDM constitutes an appealing way to improve outcomes in patients with oral anticancer therapy [5,9]. However, data from prospective, randomized studies are lacking [14,26]. An analysis published by Groenland et al. demonstrated that 38% of patients treated with 23 different oral anticancer drugs had plasma concentrations below the recommended range [14]. Previous publications made TDM-recommendations for multiple oral anticancer drugs [5,9,10]. Currently, there is a lack of appropriate methods to determine plasma concentrations for the wide variety of clinically used oral anticancer drugs. Existing methods are limited to the quantification of only a few oral anticancer drugs simultaneously, with a maximum of 17 published [27,28,29,30,31,32,33,34,35,36,37,38,39]. Each of these methods requires an individual analytical setup, which makes them suitable for a limited number of drugs only and therefore hampers the use in studies or clinical situations with a wide range of oral anticancer drugs.

The method presented in this manuscript addresses the need to cover a broad range of oral anticancer drugs as a prerequisite for systematic data on plasma concentrations of these drugs. We developed and validated a liquid chromatography-mass spectrometer (LC-MS) based method that enables the simultaneous quantification of 57 oral anticancer drugs in plasma samples, using a straightforward sample preparation. The presented method and technology (1) offer the possibility to conduct systematic studies on the impact of plasma concentrations of these drugs on clinical outcomes, (2) is suitable to implement routine drug exposure measurement for a wide variety of oral antitumor therapy prospectively, and (3) allows for the easy addition of further, newly approved compounds.

## 2. Materials and Methods

### 2.1. Chemicals and Materials

Chemical characteristics of the oral anticancer drugs which were used in the method and their respective sources are listed in Supplement Table S1. The internal standards (IS) [^2^H_4_]-everolimus and [^2^H_5_]-lenvatinib were purchased from Alsachim (Illkirch Graffenstaden, France). Next, [^2^H_4_]-olaparib was purchased from Biomol (Hamburg, Germany), and [^2^H_3_]-clopidogrel was bought from @rt molecule (Poitiers, France). Dimethyl sulfoxide (DMSO), water, acetonitrile (ACN), formic acid (FA), and methanol (MeOH) (all LC-MS grade) were purchased from VWR chemicals (Darmstadt, Germany). Pooled blank human plasma was used from the Klinische Pharmakologie Erlangen 37 (KPE37) study (described in Section 2.8).

### 2.2. Chromatography and Mass Spectrometer Equipment

The liquid chromatography-mass spectrometer system consisted of a Thermo Scientific Dionex UltiMate^TM^ 3000 with an integrated and temperature controlled autosampler (Thermo Fisher Scientific, Dreieich, Germany). An Acquity UPLC^®^ BEH C18 column (2.1 × 100 mm, 1.7 µm; Waters, Milford, CT, USA) equipped with an Acquity UPLC^®^ BEH C18 VanGuard^TM^ Pre-Column (2.1 × 5 mm, 1.7 µm; Waters, Milford, CT, USA) was used for chromatographic separation. The Q-Exactive^TM^ Focus mass spectrometer (MS) was hyphenated with a heated electrospray ionization (HESI) source (both from Thermo Fisher Scientific, Dreieich, Germany).

### 2.3. Chromatographic Conditions

Chromatographic separation was achieved using a gradient elution with water containing 0.5% (*v*/*v*) FA (eluent A) and MeOH with 0.1% (*v*/*v*) FA (eluent B). The injection volume was 5 µL. The gradient program is shown in Supplement Table S2. The flow rate was 0.5 mL/min with a total run time of 15 min. The column heater was maintained at 50 °C and the autosampler temperature was 4 °C.

### 2.4. MS Conditions

The following MS and HESI settings were maintained for all analyses: Sheath gas, auxiliary gas, and sweep gas flow rates were set to 60, 20, and 0, respectively. The spray voltage was 4000 V. Both capillary and auxiliary gas heater temperatures were set to 400 °C. High-purity nitrogen gas was used for the HESI source gases and for the bath gas of the C-trap. For MS analysis a full scan MS mode with a resolution of 17,500, a scan-range of 120–1000 *m/z* and an automatic gain control target of 1 × 10^6^ was used. The HESI source was configured in positive mode for ionization. All analytes were detected as protonated molecular ions [M + H]^+^ except for imatinib, trametinib and trifluridine, which were detected as sodium adducts [M + Na]^+^.

### 2.5. Sample Preparation

Stock solutions of all analytes and IS were prepared at a concentration of 1 mg/mL in different solvents (DMSO, ACN, MeOH) depending on their respective solubility (listed in Supplement Table S1). The stock solutions were stored in glass vials at −80 °C. Aliquots of all stock solutions were combined in ACN (volumes of individual stock solution solvents are negligible) to obtain a standard solution of all analytes with an individual concentration of 200% of the respective trough level (c_min_). Eight individual working standards were prepared by stepwise dilution of this standard solution with concentrations from 10 to 200% c_min_ (shown in Supplement Table S3). Furthermore, the four individual IS stock solutions were combined and diluted to 50 ng/mL with ACN. Calibration and validation standards were prepared by spiking 50 µL pooled human plasma with 10 µL of the respective working solution. Next, 250 µL IS solution (50 ng/mL in ACN) were added and vortexed vigorously. Subsequently, the mixtures were centrifuged at 16,220 rpm and 4 °C for 10 min using an Eppendorf 5427R centrifuge (Eppendorf; Hamburg, Germany). Then, 100 µL supernatant were transferred to 300 µL HPLC insert glass vials and diluted with 100 µL eluent (90% A/10% B). For blank samples, 50 µL pooled human plasma were combined with 10 µL ACN (instead of working solution) and prepared as described above. The calibration and validation standards were freshly prepared before every run. Patient plasma samples were prepared as calibration and validation standards (no additional processing step necessary), except for adding 10 µL ACN instead of 10 µL working standard. Before sample preparation, the frozen plasma samples were thawed in absence of light at 4 °C.

### 2.6. Patient Plasma Collections

Blood samples were collected over the entire dosing interval from patients treated with an oral anticancer drug as listed in Supplement Table S4. The study protocol was approved on 8 July 2020 (ethics committee code: 277_16 B, amendment) by the Ethics Committee of the Friedrich-Alexander-Universität Erlangen-Nürnberg and all patients provided written informed consent. A maximum of two samples at two different time points throughout the entire dosing interval were collected per patient. Treatment duration before collection was at least five half-lives (depending on the specific oral anticancer drug). The samples were collected in K3-EDTA tubes. After centrifugation at 4000 rpm and 4 °C for 20 min using an Eppendorf 5810R centrifuge (Eppendorf; Hamburg, Germany), plasma supernatants were transferred to 1.5 mL Eppendorf tubes and stored at −80 °C until analysis.

### 2.7. Data Analysis

Recording, processing and evaluation of raw data was accomplished using TraceFinder 4.1 EFS Software (Thermo Fisher Scientific; Dreieich, Germany). Validation results were analyzed with Microsoft Excel 2016. 

### 2.8. Method Validation

Validation of the MS assay and sample preparation was carried out according to the guidelines for Bioanalytical Method Validation of the US Food and Drug Administration (FDA) [40]. Blank plasma for sample preparation of the validation standards was generated and pooled from healthy female and male volunteers. Collection of plasma was performed at the Institute of Experimental and Clinical Pharmacology and Toxicology, Friedrich-Alexander-Universität Erlangen-Nürnberg (Germany) and operated under the name KPE37. Approval from the Ethics Committee of the Friedrich-Alexander-Universität Erlangen-Nürnberg was obtained on 14 September 2018 (ethics committee code: 310_18 B) and all healthy volunteers provided written informed consent. The validation range mirrored the calibration range with 10% c_min_ at steady state being the lower limit of validation (LLOV) and 200% c_min_ the upper limit of validation (ULOV).

### 2.9. Calibration Curves, Linearity, and Sensitivity

Eight-point calibration curves between LLOV and ULOV of every analyte were plotted, whereby four replicates of each point were used to assess if the coefficient of correlation (R^2^) was permanently greater than 0.995 (weighting 1/x). To meet FDA accuracy requirements, all calibration points with a concentration above the LLOV and six calibration levels overall had to be between 85 and 115% with a maximum coefficient of variation (CV) of 15%. Sensitivity was tested by determining the response of the lowest non-zero standard of the calibration standards compared to the response of a blank sample (prepared with IS). The acceptance criterion was fulfilled if analyte response in the blank sample was five times lower compared to the response in the LLOV.

### 2.10. Accuracy and Precision

Accuracy and precision were determined using five replicates of six different concentration levels. Pooled human plasma samples were spiked to concentrations as shown in Supplement Table S5. Five replicates of all validation standards were analyzed for intraday accuracy and precision on one day. For assessment of interday accuracy and precision, five replicates of all validation standards were analyzed by performing single runs on five different days. The required FDA limits of accuracy and precision are 85–115% and ≤15% for the CV, except for LLOV. Here, the limits of accuracy, precision, and CV are 80–120% and ≤20%, respectively.

### 2.11. Stability

Assessment of freeze-thaw stability was performed by preparing four validation standards (LLOV, 2 × LLOV, middle of validation limit (MOV), ULOV) that were stored at −80 °C and thawed three times at 4 °C. After thawing, samples were frozen for 24 h between cycles. Bench-top stability was evaluated at ambient conditions with or without light for 24 h. Stability at 4 °C was tested by storing the four validation standards in a fridge at 4 °C for 24 h and 6 weeks. Similarly, stability was analyzed after incubation at 56 °C for 1 h with an Eppendorf Thermomixer compact (Eppendorf; Hamburg, Germany). Autosampler stability was assessed by measuring ready for injection samples after being kept in the autosampler at 4 °C for 24 and 72 h, respectively. Long-term stability was tested after storing three replicates of the four validation standards at −20 °C and −80 °C for 1, 2, and 3 months. Stability was accepted if measured values were within the FDA range, i.e., ≤15% for 2 × LLOV, MOV and ULOV, and ≤20% for LLOV, of their nominal concentration.

### 2.12. Recovery and Matrix Effect

For recovery investigation three replicates of five validation standards (LLOV, 2 × LLOV, low limit of validation (LOV), MOV, and ULOV) were prepared by spiking working solutions before and after plasma protein precipitation into pooled human plasma. Recovery was defined as the ratio of pre-extraction to post-extraction addition signal intensity. For matrix effect investigation, working solutions were spiked into water (instead of human plasma) before the extraction was performed. Ratios between the analyte responses of post-extraction addition into pooled human plasma and into water informed about the extent of matrix effects.

### 2.13. Selectivity

Selectivity was investigated by analyzing six blank plasma samples from healthy individuals and was accepted if the samples were free of interferences at the retention time(s) of the analyte(s) and IS. Acceptance criteria for any interference was a signal response ≤ 20% of that of the LLOV and ≤5% of that of the IS.

### 2.14. Carryover

Carryover was assessed by injecting two blank samples (without IS) directly after running the highest calibration level. To fulfill the acceptance criteria, signal intensities had to be below 20% of the peak area of an LLOV reference and less than 5% for IS.

### 2.15. Dilution Integrity

Dilution integrity was analyzed by preparing pooled human plasma samples spiked with analytes to a four-fold concentration than the ULOV, followed by an eight-fold dilution of this sample with pooled human plasma.

## 3. Results

**Figure 1:**
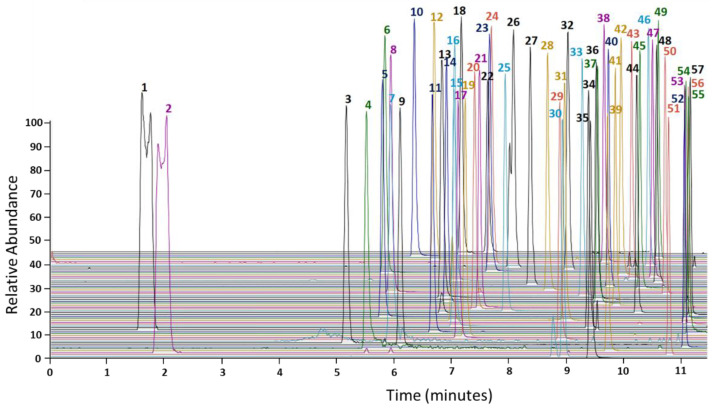
Overview of all 57 separated oral anticancer drugs. Combined representation of individual chromatograms. 100% relative abundance corresponds to the maximum peak height of each compound. Lenalidomide (1), trifluridine (2), pomalidomide (3), thalidomide (4), ribociclib (5), brigatinib (6), panobinostat (7), gefitinib (8), rucaparib (9), abemaciclib (10), lorlatinib (11), afatinib (12), imatinib (13), lenvatinib (14), vandetanib (15), crizotinib (16), niraparib (17), dacomitinib (18), neratinib (19), palbociclib (20), osimertinib (21), ruxolitinib (22), bosutinib (23), anagrelide (24), pazopanib (25), axitinib (26), dasatinib (27), erlotinib (28), olaparib (29), vinorelbine (30), sunitinib (31), binimetinib (32), idelalisib (33), larotrectinib (34), vismodegib (35), lapatinib (36), nilotinib (37), alectinib (38), tivozanib (39), encorafenib (40), nintedanib (41), cabozantinib (42), cobimetinib (43), ponatinib (44), enzalutamide (45), apalutamide (46), ceritinib (47), dabrafenib (48), abiraterone (49), ibrutinib (50), venetoclax (51), sonidegib (52), trametinib (53), sorafenib (54), midostaurin (55), vemurafenib (56), regorafenib (57).

A LC-MS based method for the separation and quantification of 57 oral anticancer drugs was developed and fully validated. Different compositions and pH values (1.96–5.51) of mobile phases (ACN, MeOH, H_2_O) were tested for chromatographic separation. Optimal separation of all 57 oral anticancer drugs was achieved using a gradient of water containing 0.5% FA and MeOH containing 0.1% FA (Figure 1). Previous publications reported excellent results for the chromatographic separation of oral antitumor drugs via FA as a mobile phase additive [33,36,37,41]. Thus, FA was chosen for ionic strength modification. Four IS were chosen based on their retention times and in-house availability. Analytes were split in four groups and normalized to the IS with the closest retention time. The validation was performed according to the FDA guidelines and involved the following points (Section 3.1, Section 3.2, Section 3.3, Section 3.4 and Section 3.5).

### 3.1. Calibration Curves, Linearity, Carryover, Selectivity, and Sensitivity

All 57 oral antitumor drugs fulfilled the FDA acceptance criteria. Correlation coefficients (R^2^) were determined for all analytes and were permanently ≥0.995. Carryover was observed in eight analytes with values from 0.03% (enzalutamide) to 12.7% (midostaurin). Selectivity investigations showed that after injection of six blank plasma samples from healthy individuals, there was no interfering signal with a response of ≥20% of LLOV and ≥5% of IS detectable. Sensitivity results fulfilled the acceptance criteria, with the exception of the two lowest calibration levels of panobinostat (48.9% at LLOV, 23.4% at 2 × LLOV). Thus, panobinostat fulfilled acceptance criteria from LOV to ULOV. All results are shown in Supplement Table S5.

### 3.2. Accuracy and Precision

FDA acceptance criteria were fulfilled for all 57 oral antitumor drugs with an eight-point calibration curve, with the exceptions of the LLOV of neratinib (accuracy 72.6%) and panobinostat (CV 20.2%). Intra- and interday accuracy and precision at different concentration levels (LLOV, 2 × LLOV, LOV, MOV, HOV (high limit of validation), ULOV) are shown in Supplement Table S5. At the LLOV, intraday accuracies varied between 82.7% (afatinib) and 117.7% (sunitinib) with CVs of ≤13.0%. Accuracies at other concentration levels ranged between 86.3% (ribociclib) and 114.0% (neratinib) with a CV of ≤9.3%. Interday accuracies at the LLOV ranged between 81.7% (trifluridine) and 118.5% (dasatinib) with a CV of ≤5.2%. Thus, all validation standards fulfilled the acceptance criteria for interday accuracy with values between 89.6% (crizotinib) and 114.5% (neratinib) and a CV of ≤2.9%.

### 3.3. Recovery and Matrix Effect

Results of recovery and matrix effect investigations are listed in Supplement Table S5. Venetoclax showed the lowest mean recovery with a percentage of 89.0%, compared to brigatinib with the highest mean recovery of 98.0%. All analytes, except for ponatinib, showed ion enhancement. Most remarkable were the ion enhancement effects for abemaciclib, bosutinib, brigatinib, and imatinib, with values of 1131.0, 165.1, 1855.0 and 153.0%, respectively. Ion enhancement for other analytes was between 0.3% (binimetinib) and 43.3% (neratinib). Ponatinib on the other hand experienced ion suppression of −5.0%. 

### 3.4. Stability

Detailed stability test results are shown in Supplement Tables S6 and S7. After the freeze-thaw stability investigation, all 57 oral antitumor drugs fulfilled the acceptance criteria, except for the LLOVs of crizotinib, neratinib, pomalidomide, vismodegib, and thalidomide. Short-term stability investigations fulfilled the acceptance criteria for 32 compounds and minor stability issues were indicated for 25 compounds. After incubation at 56 °C for one hour, none of the analytes were stable. All compounds fulfilled the acceptance criteria when stored at 4 °C for 24 h, except for apalutamide, neratinib, panobinostat, ribociclib, thalidomide, and the LLOVs of axitinib, bosutinib, and dasatinib. Autosampler stability tests results fulfilled the acceptance criteria except for at least one concentration level of imatinib, lenalidomide, and the LLOVs of larotrectinib, neratinib, panobinostat, and vinorelbine. Analytes were stable at −20 °C and −80 °C for 1, 2, and 3 months, except for afatinib, alectinib, crizotinib, neratinib, panobinostat, sunitinib, thalidomide, and trametinib at the end of long-term stability investigations. Another 15 analytes showed negligible deviations. After six weeks at 4 °C, 31 compounds fulfilled the acceptance criteria and 26 analytes showed minor accuracy deviations.

### 3.5. Dilution Integrity

For concentrations four times higher than ULOV, 49 of the oral anticancer drug dilutions showed accuracies (85.4–115.0%) within the acceptance criteria. Brigatinib, dacomitinib, encorafenib, osimertinib, pazopanib, regorafenib, rucaparib, and trifluridine did not fulfill the acceptance criteria. Detailed results of dilution integrity investigations are shown in Supplement Table S5.

### 3.6. Patient Samples

**Figure 2:**
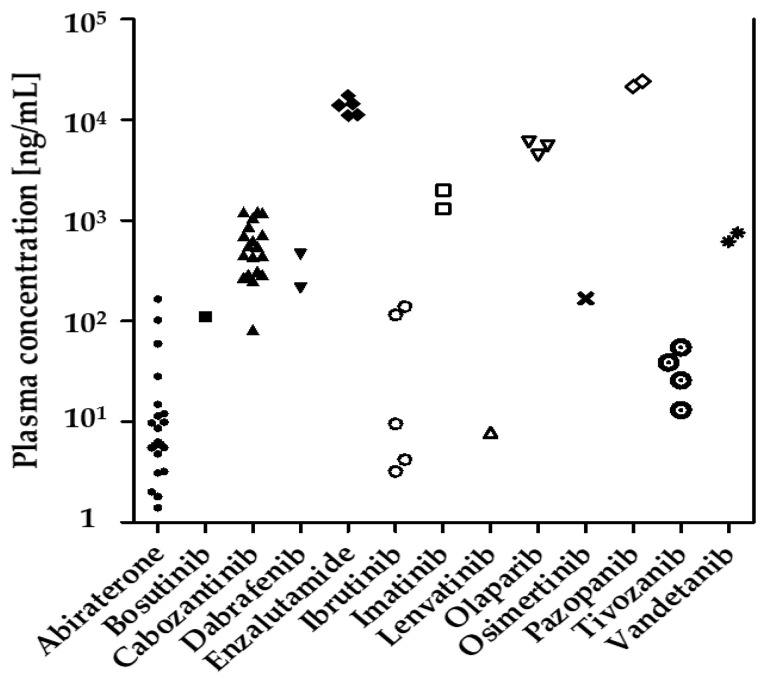
Overview of all measured concentrations of oral antitumor drugs in 71 plasma samples collected from 39 patients.

**Figure 3:**
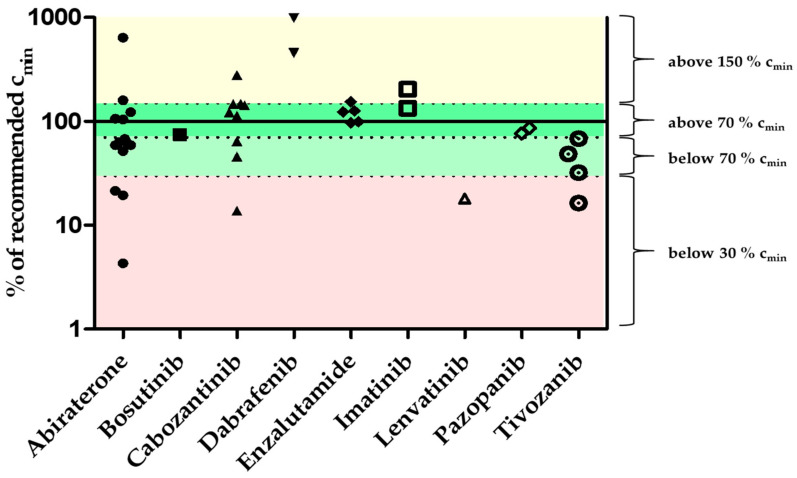
Overview of measured oral antitumor drug concentrations in patient plasma samples collected ±4 h of trough level. The percentages of the plasma concentrations were calculated using the respective c_min_ value (equivalent to 100%). Colored areas indicate whether the sample is below 30% (red), below 70% (light green), above 70% (green), or above 150% (yellow) of the respective c_min_ value.

A total of 71 blood samples from 39 patients treated with 14 different oral anticancer drugs were collected (see Supplement Table S4 for details). All patients were recruited from independent outpatient clinics associated with the Comprehensive Cancer Center of the University Hospital Erlangen (CCC Erlangen-EMN). A summary of the results is shown in Table 1. An overview of all plasma concentrations is shown in Figure 2. Of 71 blood samples, 41 were taken ±4 h of trough level (c_min_). The measured concentrations of these 41 samples are presented as % c_min_ in Figure 3.

## 4. Discussion

The present work constitutes a new validated multi-drug MS method for simultaneous determination of 57 oral antitumor drugs in human plasma. Recently, various research groups aimed at the development of methods for the simultaneous quantification of oral antitumor drugs [27,28,29,30,31,32,33,34,35,36,37,38,39]. The developed methods targeted a limited number of oral antitumor drugs, which is not representative for the broad range of oral anticancer drugs that is used in clinical routine nowadays. To the best of our knowledge, the highest number of compounds to date was included in a method by Merienne et al. [28], quantifying 17 kinase inhibitors simultaneously. This limitation is likely a consequence of the type of MS commonly applied for targeted methods, i.e., a triple quadrupole (QQQ)-MS. For targeted analyses, QQQ-MS are operated in a multiple ion monitoring mode, during which a parent ion of a specific analyte is isolated and fragmented. Parent ion and fragments are specific for the targeted analyte and thus permit identification with a high level of certainty. However, these ions and their optimal MS parameters have to be determined for each individual analyte during method development, which is very time consuming and complex for a high number of compounds. Furthermore, this approach only allows for the detection of a predefined number of compounds and any other present molecules that might be interesting remain undetected.

In the present work we circumvented this limitation by employing an Orbitrap MS. This high mass resolution MS enables the analyte identification based on a highly resolved mass to charge ratio and fragmentation is not necessarily required. Using this analytical hardware, it was possible to develop a method for the simultaneous quantification of 57 oral antitumor drugs, which is to the best of our knowledge the highest number of drugs covered by a single method. In addition, our method offers the advantage of being easily extendable to include further oral antitumor drugs and drug metabolites of interest. This is possible due to the full scan mode in which the Orbitrap MS was operated in this study. Furthermore, acquisition in full scan mode allows for retrospective detection of metabolites that are not the focus of this research, but might be in the future. In previous literature, one other method applied an Orbitrap MS for the quantification of oral antitumor drugs, however, only six kinase inhibitors were quantified simultaneously [39].

Validation results indicated accordance with the requirements of the FDA guidelines [40]. For concentrations within the calibration range, only neratinib and panobinostat indicated minor deviations from the FDA guideline acceptance criteria, which were limited to the LLOV for neratinib and LLOV and 2 × LLOV for panobinostat. Consequently, the calibration curves of these two drugs were truncated by removing these concentration levels. Since the remaining calibration curves included six and seven points, the FDA requirements are met. Furthermore, the substances brigatinib, dacomitinib, encorafenib, osimertinib, pazopanib, regorafenib, rucaparib, and trifluridine did not meet the FDA acceptance criteria for dilution integrity after eight-fold dilution of a highly concentrated plasma sample. Thus, quantification of 49 of the 57 oral antitumor drugs is possible for concentrations between 200 and 800% c_min_ after adequate dilution.

Previous publications reported stability issues for some oral anticancer drugs. For instance, osimertinib and afatinib concentrations decreased under ambient conditions [29,41]. These findings are in accordance with the current results, in which short-term stability investigations (24 h, room temperature, with or without light) of afatinib and osimertinib showed concentrations ranging between 64.3–92.5% and 60.5–75.7%, respectively. Furthermore, light sensitivity has been reported in literature for axitinib and dabrafenib [42,43]. This was not in accordance with our results since no losses of concentrations below the acceptance limit (<85%) were detected. Long-term investigation at −80 °C demonstrated stable concentrations of all oral antitumor drugs (Supplement Table S7). All stability findings highlight the need for an adequate pre-analytical sample handling. Therefore, all samples should be transported and processed as soon as possible after blood collection and ideally stored at −80 °C until measurement. Preparation time for samples containing afatinib or osimertinib at ambient conditions should be reduced to a minimum.

The applicability of the method to a real-life clinical setting was subsequently assessed in a pilot study, including patient plasma samples from the CCC Erlangen-EMN. 71 plasma samples were collected, of which 41 samples were taken ±4 h of trough level according to the patient’s statement. In line with the literature, the results demonstrate a significant interindividual variability of the plasma concentrations of the respective antitumor drugs (Figure 2). For example, the highest interindividual variability (187.3%) was observed in patients treated with abiraterone. Of 22 collected samples, 14 (64%) were taken at ±4 h trough level. The plasma concentrations at ±4 h of trough level were below 30% c_min_ for three samples, nine were within 30–150% c_min_ and two were >150% c_min_. The average concentration of the plasma samples at ±4 h of trough level was 10.2 ng/mL (range 0.4–59.5 ng/mL, CV 140.8%, Figure 3). Van Nuland and colleagues found an average plasma concentration of abiraterone of 9.3 ng/mL (2.0–49.8 ng/mL, CV 70.0%) in 244 plasma samples, which is similar to the findings presented in this work [44]. In line with previous publications (see [5,9,14] for recent reviews) our results indicate that drug exposure measurements may reveal the need for therapy adjustment in a significant proportion of patients. In real-world clinical setting blood sampling at trough level is challenging. Consequently, extrapolation of measured plasma concentrations via pharmacokinetic modeling may be useful to calculate trough level plasma concentrations as demonstrated in previous publications [5,45,46].

The high interindividual variability in plasma concentrations found in the present work and in previous publications [5,9,10,14] is likely due to multiple factors, such as varying time between intake of medication and blood sampling, variable patient adherence [6,16], and interactions with food and/or concomitant medications (e.g., acid reducing agents) [6,17,18,19]. Recently, we reported higher patient satisfaction, reduced side effects and medication errors under treatment with many new oral antitumor drugs when an intensified clinical pharmacological/pharmaceutical care program is applied on top of routine clinical care [8]. The method presented in this work allows for further studies on the association of plasma concentrations with clinical outcome. This drug exposure measurement would be a significant step towards implementation of TDM of oral antitumor drugs in clinical routine for which a highly flexible and reliable method, as presented here, is a prerequisite.

## 5. Conclusions

A variety of factors influence the pharmacokinetics of oral antitumor drugs and consequently clinical outcome. Routine measurement of plasma concentrations via TDM has not been implemented for most oral antitumor drugs yet. This is most likely due to the requirement of applying multiple analytical methods to cover the broad range of approved drugs, which has deemed routine drug exposure measurement too labor-intensive and time-consuming to date. The new method presented here employs a high resolution Orbitrap MS in full scan mode in order to overcome this hurdle. The high mass resolution allows for the simultaneous measurement of 57 oral antitumor drugs without the need for a change of analytical hardware. It also provides an opportunity to easily expand the included analytes with newly approved oral antitumor drugs and metabolites of clinical relevance. Consequently, the presented method sets a foundation for future studies to investigate the putative benefits of routine drug exposure measurement for a wide range of oral antitumor drugs.

## Figures and Tables

**Table 1:** Summary of results from 71 plasma samples of patients treated with an oral anticancer drug.

Oral Antitumor Drug	Number of Samples/Patients	Samples at ±4 h Trough Level	Median Concentration of All Samples [% of r.c.]	Min–Max Concentration [% of r.c.]	Interpatient Coefficient of Variation [%]
Abiraterone	22/12	14	229.0	4.3–1784.9	187.3
Axitinib	1/1	0	-	6.0	-
Bosutinib	1/1	1	-	74.7	-
Cabozantinib *	19/11	9	153.3	13.8–207.2	58.5
Dabrafenib	2/1	2	722.3	455.8–988.8	-
Enzalutamide	5/3	5	120.1	97.1–154.2	19.5
Ibrutinib	4/2	0	110.1	4.8–211.4	110.0
Imatinib	2/2	2	168.9	133.8–204.1	29.4
Lenvatinib	2/1	2	-	n.d.–18.0	-
Olaparib	4/2	0	287.6	n.d.–325.5	51.6
Osimertinib	1/1	0	-	101.1	-
Pazopanib	2/1	2	81.5	76.6–86.5	-
Tivozanib	4/2	4	41.4	16.4–68.3	53.8
Vandetanib	2/1	0	86.7	78.2–95.3	-

n.d. = not detectable, r.c. = respective c_min_, * For the calculations of the concentrations in %, dose-individual c_min_ values were used. For dosages of 20, 40 and 60 mg, the following c_min_ values were used: 197.1, 394.3 and 591.4 ng/mL, respectively.

## Data Availability

The data presented in this study are available on request from the corresponding authors. The data are not publicly available due to ethical restrictions.

## Supplementary Materials

The following are available online at https://www.mdpi.com/article/10.3390/cancers13246329/s1, Table S1: Summary of c_min_-values, LC-MS settings and properties of the oral anticancer drugs; Table S2: Gradient elution program; Table S3: Overview of the concentrations of eight validation standards and the corresponding internal standards of each oral antitumor drug; Table S4: Oral antitumor therapy information of individual patients and measured plasma concentrations; Table S5: Validation results of the 57 oral antitumor drugs following the FDA guidelines; Table S6: Validation results of stability tests; Table S7: Long-term stability test results.
