# Supplementary material for: An Easily Expandable Multi-Drug LC-MS Assay for the Simultaneous Quantification of 57 Oral Antitumor Drugs in Human Plasma
Source: Cancers (Basel). 2021 Dec 16;13(24):6329. doi: 10.3390/cancers13246329 (PMC8699473; doi:10.3390/cancers13246329)
Supplement: Supplementary file 1 [file cancers-13-06329-s001.zip › cancers-1480681_sup-done_revisionv2.pdf]

cancers

MDPI

An easily expandable multi-drug LC-MS assay for the simultaneous quantification of 57 oral antitumor drugs in human plasma

Niklas Kehl [1,2], Katja Schlichtig [1,2], Pauline Dürr [1,2,3], Laura Bellut [2,4], Frank Dörje [2,3], Rainer Fietkau [2,5], Marianne Pavel [2,6], Andreas Mackensen [2,7], Bernd Wullich [2,4], Renke Maas [1,2], Martin F. Fromm [1,2], Arne Gessner [1,2,*,†] and R. Verena Taudte [1,2,*,†]

Table S1. Summary of c_{min}-values[#], LC-MS settings and properties of the oral anticancer drugs.

Oral anticancer drug	c_{min} [ng/mL] [Reference]	Structural formula	Monoisotopic mass [Da]	Detected ion m/z	Detected species	Calculated ion m/z	Difference between detected and calculated ion [ppm]	Validated range [ng/mL]	Lower limit of validation [ng/mL]	Correlation coefficient [R²]	Retention time [min]	Dissolved in	Purchased from
**Abemaciclib**	181 [1,2]	$C_{27}H_{32}F_2N_8$	506.2718	507.2791	+ H	507.2791	0.05	20-400	20.00	0.9980	6.3	DMSO	Absource (Munich, Germany)
**Abiraterone**	9.3 [2,3]	$C_{24}H_{31}NO$	349.2406	350.2478	+ H	350.2478	-0.12	1-20	1.00	0.9987	10.5	DMSO	Absource (Munich, Germany)
**Afatinib**	14.4 [2,4,5]	$C_{24}H_{25}ClFN_5O_3$	485.1630	486.1703	+ H	486.1703	0.06	1.5-30	1.50	0.9993	6.7	DMSO	LC Laboratories (Woburn, USA)
**Alectinib**	572 [2,5,6]	$C_{30}H_{34}N_4O_2$	482.2682	483.2755	+ H	483.2755	0.10	60-1200	60.00	0.9992	9.8	DMSO	LC Laboratories (Woburn, USA)
**Anagrelide**	1.5* [7]	$C_{10}H_7Cl_2N_3O$	254.9966	256.0039	+ H	256.0039	0.03	0.75-15	0.75	0.9996	7.6	DMSO	Absource (Munich, Germany)
**Apalutamide**	3700 [2,8]	$C_{21}H_{15}F_4N_5O_2S$	477.0883	478.0955	+ H	478.0955	-0.07	375-7500	375.00	0.9997	10.5	DMSO	Absource (Munich, Germany)
**Axitinib**	5 [2,5,9]	$C_{22}H_{18}N_4OS$	386.1201	387.1274	+ H	387.1274	-0.02	0.5-10	0.50	0.9964	8.0	MeOH	Merck (Darmstadt, Germany)
**Binimetinib**	53.3 [2,10]	$C_{17}H_{15}BrF_2N_4O_3$	440.0296	441.0368	+ H	441.0368	-0.08	5-100	5.00	0.9972	9.0	DMSO	LC Laboratories (Woburn, USA)
**Bosutinib**	147 [2,5,11]	$C_{26}H_{29}Cl_2N_5O_3$	529.1647	530.1720	+ H	530.1720	-0.04	15-300	15.00	0.9952	7.6	DMSO/ ACN/ MeOH (1:1:1)	LC Laboratories (Woburn, USA)
**Brigatinib**	520 [2,12]	$C_{29}H_{39}ClN_7O_2P$	583.2591	584.2664	+ H	584.2664	-0.02	55-1100	55.00	0.9972	5.9	DMSO	LC Laboratories (Woburn, USA)

**Cabozantinib**	1380 [2,5,13]	$C_{28}H_{24}FN_3O_5$	501.1700	502.1773	+ H	502.1773	0.05	150-3000	150.00	0.9982	9.9	DMSO	LC Laboratories (Woburn, USA)
**Ceritinib**	871 [2,5,14]	$C_{28}H_{36}ClN_5O_3S$	557.2227	558.2300	+ H	558.2300	-0.03	90-1800	90.00	0.9991	10.6	DMSO	LC Laboratories (Woburn, USA)
**Cobimetinib**	127 [2,5,15]	$C_{21}H_{21}F_3IN_3O_2$	531.0631	532.0703	+ H	532.0703	-0.07	12.5-250	12.50	0.9997	10.0	DMSO	LC Laboratories (Woburn, USA)
**Crizotinib**	274 [2,5,16]	$C_{21}H_{22}Cl_2FN_5O$	449.1185	450.1258	+ H	450.1258	-0.04	25-500	25.00	0.9952	7.0	MeOH	Merck (Darmstadt, Germany)
**Dabrafenib**	46.6 [2,5,17]	$C_{23}H_{20}F_3N_5O_2S_2$	519.1011	520.1083	+ H	520.1083	-0.05	5-100	5.00	0.9991	10.6	DMSO	LC Laboratories (Woburn, USA)
**Dacomitinib**	73.1 [2,18]	$C_{24}H_{25}ClFN_5O_2$	469.1681	470.1754	+ H	470.1754	0.09	7.5-150	7.50	0.9992	7.1	MeOH	Biomol (Hamburg, Germany)
**Dasatinib**	2.61 [2,5,19]	$C_{22}H_{26}ClN_7O_2S$	487.1557	488.1630	+ H	488.1630	0.00	1-20	1.00	0.9954	8.3	DMSO	LC Laboratories (Woburn, USA)
**Encorafenib**	23 [2,20]	$C_{22}H_{27}ClFN_7O_4S$	539.1518	540.1591	+ H	540.1591	0.08	2.5-50	2.50	0.9992	9.6	MeOH	Biomol (Hamburg, Germany)
**Enzalutamide**	11400 [2,21]	$C_{21}H_{16}F_4N_4O_2S$	464.0930	465.1003	+ H	465.1003	0.03	1125-22500	1125.00	0.9998	10.2	DMSO	Absource (Munich, Germany)
**Erlotinib**	1011 [2,5,22]	$C_{22}H_{23}N_3O_4$	393.1689	394.1761	+ H	394.1761	-0.08	100-2000	100.00	0.9961	8.6	DMSO	LC Laboratories (Woburn, USA)
**Gefitinib**	291 [2,5,23]	$C_{22}H_{24}ClFN_4O_3$	446.1521	447.1594	+ H	447.1594	0.06	30-600	30.00	0.9987	5.9	DMSO	Merck (Darmstadt, Germany)
**Ibrutinib**	66* [2,5,24]	$C_{25}H_{24}N_6O_2$	440.1961	441.2034	+ H	441.2034	0.11	7.5-150	7.50	0.9996	10.7	DMSO	LC Laboratories (Woburn, USA)
**Idelalisib**	318 [2,5,25]	$C_{22}H_{18}FN_7O$	415.1557	416.1630	+ H	416.1630	0.09	40-800	40.00	0.9957	9.2	DMSO	LC Laboratories (Woburn, USA)
**Imatinib**	1193 [2,5,26]	$C_{29}H_{31}N_7O$	493.2590	516.2482	+ Na	516.2482	-0.05	100-2000	100.00	0.9976	6.8	MeOH	Santa Cruz (Heidelberg, Germany)
**Lapatinib**	780 [2,5,27]	$C_{29}H_{26}ClFN_4O_4S$	580.1347	581.1420	+ H	581.1420	-0.01	80-1600	80.00	0.9980	9.7	DMSO	LC Laboratories (Woburn, USA)
**Larotrectinib**	5.14 [2]	$C_{21}H_{22}F_2N_6O_2$	428.1772	429.1845	+ H	429.1845	-0.01	2.5-50	2.50	0.9970	9.3	MeOH	Biomol (Hamburg, Germany)
**Lenalidomide**	13 [28]	$C_{13}H_{13}N_3O_3$	259.0957	260.1030	+ H	260.1030	0.13	1.5-30	1.50	0.9994	1.7	DMSO	Merck (Darmstadt, Germany)
**Lenvatinib**	51.5 [2,5,29]	$C_{21}H_{19}ClN_4O_4$	426.1095	427.1168	+ H	427.1168	0.10	5-100	5.00	0.9977	6.9	DMSO	LC Laboratories (Woburn, USA)
**Lorlatinib**	150 [2,30]	$C_{21}H_{19}FN_6O_2$	406.1554	407.1626	+ H	407.1626	-0.07	15-300	15.00	0.9997	6.7	MeOH	Biomol (Hamburg, Germany)

**Midostaurin**	1122,8 [2,31]	$C_{35}H_{30}N_4O_4$	570.2267	571.2340	+ H	571.2340	0.03	100-2000	100.00	0.9988	11.2	DMSO	Absource (Munich, Germany)
**Neratinib**	52.6 [32]	$C_{30}H_{29}ClN_6O_3$	556.1990	557.2062	+ H	557.2062	-0.08	11-110	5.50	0.9972	7.2	DMSO	LC Laboratories (Woburn, USA)
**Nilotinib**	1165 [2,5,33]	$C_{28}H_{22}F_3N_7O$	529.1838	530.1911	+ H	530.1911	0.06	120-2400	120.00	0.9999	9.5	MeOH:H_2O (4:1)	LC Laboratories (Woburn, USA)
**Nintedanib**	13.1 [2,5,34]	$C_{31}H_{33}N_5O_4$	539.2533	540.2605	+ H	540.2605	-0.06	1.5-30	1.50	0.9994	10.0	DMSO	LC Laboratories (Woburn, USA)
**Niraparib**	649 [2]	$C_{19}H_{20}N_4O$	320.1637	321.1710	+ H	321.1710	0.04	65-1300	65.00	0.9994	7.1	DMSO	LC Laboratories (Woburn, USA)
**Olaparib**	1570 [2,35]	$C_{24}H_{23}FN_4O_3$	434.1754	435.1827	+ H	435.1827	0.01	150-3000	150.00	0.9973	8.8	DMSO	LC Laboratories (Woburn, USA)
**Osimertinib**	166 [2,5,36]	$C_{28}H_{33}N_7O_2$	499.2696	500.2769	+ H	500.2768	0.10	17.5-350	17.50	0.9990	7.5	DMSO	LC Laboratories (Woburn, USA)
**Palbociclib**	61 [2,5,37]	$C_{24}H_{29}N_7O_2$	447.2383	448.2456	+ H	448.2455	0.11	6-120	6.00	0.9997	7.4	DMSO	Merck (Darmstadt, Germany)
**Panobinostat**	1.05 [2,38]	$C_{21}H_{23}N_3O_2$	349.1790	350.1863	+ H	350.1863	-0.01	5-20	1.00	0.9983	5.9	DMSO	LC Laboratories (Woburn, USA)
**Pazopanib**	24000 [2,5,39]	$C_{21}H_{23}N_7O_2S$	437.1634	438.1707	+ H	438.1707	0.07	52-1040	52.00	0.9966	7.9	DMSO	Merck (Darmstadt, Germany)
**Pomalidomide**	10.9 [40]	$C_{13}H_{11}N_3O_4$	273.0750	274.0822	+ H	274.0822	-0.12	4-80	4.00	0.9991	5.1	DMSO	Absource (Munich, Germany)
**Ponatinib**	34 [2,5,41]	$C_{29}H_{27}F_3N_6O$	532.2198	533.2271	+ H	533.2271	-0.04	3.5-70	3.50	0.9993	10.2	DMSO	LC Laboratories (Woburn, USA)
**Regorafenib**	1400 [2,5,42]	$C_{21}H_{15}ClF_4N_4O_3$	482.0769	483.0842	+ H	483.0842	0.09	140-2800	140.00	0.9992	11.1	DMSO	LC Laboratories (Woburn, USA)
**Ribociclib**	732 [2,43]	$C_{23}H_{30}N_8O$	434.2543	435.2615	+ H	435.2615	-0.08	75-1500	75.00	0.9963	5.8	DMSO	LC Laboratories (Woburn, USA)
**Rucaparib**	1754 [2,44]	$C_{19}H_{18}FN_3O$	323.1434	324.1507	+ H	324.1507	0.10	175-3500	175.00	0.9994	6.1	DMSO	LC Laboratories (Woburn, USA)
**Ruxolitinib**	11.4 [2,45]	$C_{17}H_{18}N_6$	306.1593	307.1666	+ H	307.1666	0.10	1.25-25	1.25	0.9994	7.6	DMSO	LC Laboratories (Woburn, USA)
**Sonidegib**	890 [46]	$C_{26}H_{26}F_3N_3O_3$	485.1926	486.1999	+ H	486.1999	0.00	100-2000	100.00	0.9990	11.0	DMSO	LC Laboratories (Woburn, USA)
**Sorafenib**	3750 [2,5,47]	$C_{21}H_{16}ClF_3N_4O_3$	464.0863	465.0936	+ H	465.0936	0.05	375-7500	375.00	0.9994	11.1	DMSO	Absource (Munich, Germany)
**Sunitinib**	51.6 [2,5,22]	$C_{22}H_{27}FN_4O_2$	398.2118	399.2191	+ H	399.2191	0.05	5-100	5.00	0.9963	9.0	MeOH	Merck (Darmstadt, Germany)
**Thalidomide**	600 [48]	$C_{13}H_{10}N_2O_4$	258.0641	259.0713	+ H	259.0713	-0.13	60-1200	60.00	0.9994	5.5	DMSO	Absource (Munich, Germany)

**Tivozanib**	80 [2,49]	$C_{22}H_{19}ClN_4O_5$	454.1044	455.1117	+ H	455.1117	0.06	8-160	8.00	0.9997	9.7	DMSO	Absource (Munich, Germany)
**Trametinib**	12.1 [2,5,50]	$C_{26}H_{23}FIN_5O_4$	615.0779	638.0671	+ Na	638.0671	0.00	2.5-50	2.50	0.9977	11.0	DMSO	BIOZOL (Munich, Germany)
**Trifluridine**	1135* [51]	$C_{10}H_{11}F_3N_2O_5$	296.0620	319.0512	+ Na	319.0512	-0.08	125-2500	125.00	0.9954	2.0	DMSO	Absource (Munich, Germany)
**Vandetanib**	795 [2,5,52]	$C_{22}H_{24}BrFN_4O_2$	474.1067	475.1139	+ H	475.1139	-0.10	80-1600	80.00	0.9962	7.0	DMSO	LC Laboratories (Woburn, USA)
**Vemurafenib**	39000 [2,5,53]	$C_{23}H_{18}ClF_2N_3O_3S$	489.0725	490.0798	+ H	490.0798	-0.05	100-2000	100.00	0.9979	11.1	DMSO	LC Laboratories (Woburn, USA)
**Venetoclax**	520 [2,54]	$C_{45}H_{50}ClN_7O_7S$	867.3181	868.3254	+ H	868.3254	0.03	55-1100	55.00	0.9987	10.8	DMSO	LC Laboratories (Woburn, USA)
**Vinorelbine**	2.6 [55]	$C_{45}H_{54}N_4O_8$	778.3942	779.4014	+ H	779.4014	-0.05	6-120	6.00	0.9954	9.0	DMSO	Absource (Munich, Germany)
**Vismodegib**	11367 [2]	$C_{19}H_{14}Cl_2N_2O_3S$	420.0102	421.0175	+ H	421.0175	0.01	1150-23000	1150.00	0.9980	9.4	DMSO	LC Laboratories (Woburn, USA)

minimum steady-state blood plasma concentration after multiple dose administration. * calculated based on c_{max}

Table S2. Gradient elution program.

Time [min]	Eluent A[1] [%]	Eluent B[2] [%]
0	90	10
9	2	98
11	2	98
11.5	90	10
15	90	10

[1] Eluent A: water containing 0.5% (vol/vol) FA, [2] Eluent B: methanol containing 0.1% (vol/vol) FA.

Table S3. Overview of the concentrations of eight validation standards and the corresponding internal standards of each oral antitumor drug.

Oral anticancer drug	STD 1 (ULOV)	STD 2	STD 3 (HOV)	STD 4 (MOV)	STD 5	STD 6 (LOV)	STD 7 (2xLLOV)	STD 8 (LLOV)	IS
Abemaciclib	400	320	256	200	160	100	40	20	[$^2H_5$]-lenvatinib (RT 6.8 min)
Abiraterone	20	16	12.8	10	8	5	2	1	[$^2H_3$]-clopidogrel (RT 9.7 min)
Afatinib	30	24	19.2	15	12	7.5	3	1.5	[$^2H_5$]-lenvatinib (RT 6.8 min)
Alectinib	1200	960	768	600	480	300	120	60	[$^2H_3$]-clopidogrel (RT 9.7 min)
Anagrelide	15	12	9.6	7.5	6	3.75	1.5	0.75	[$^2H_5$]-lenvatinib (RT 6.8 min)
Apalutamide	7500	6000	4800	3750	3000	1875	750	375	[$^2H_3$]-clopidogrel (RT 9.7 min)
Axitinib	10	8	6.4	5	4	2.5	1	0.5	[$^2H_4$]-olaparib (RT 8.8 min)
Binimetinib	100	80	64	50	40	25	10	5	[$^2H_4$]-olaparib (RT 8.8 min)
Bosutinib	300	240	192	150	120	75	30	15	[$^2H_5$]-lenvatinib (RT 6.8 min)
Brigatinib	1100	880	704	550	440	275	110	55	[$^2H_5$]-lenvatinib (RT 6.8 min)
Cabozantinib	3000	2400	1920	1500	1200	750	300	150	[$^2H_3$]-clopidogrel (RT 9.7 min)
Ceritinib	1800	1440	1152	900	720	450	180	90	[$^2H_3$]-clopidogrel (RT 9.7 min)
Cobimetinib	250	200	160	125	100	62.5	25	12.5	[$^2H_3$]-clopidogrel (RT 9.7 min)
Crizotinib	500	400	320	250	200	125	50	25	[$^2H_5$]-lenvatinib (RT 6.8 min)
Dabrafenib	100	80	64	50	40	25	10	5	[$^2H_3$]-clopidogrel (RT 9.7 min)
Dacomitinib	150	120	96	75	60	37.5	15	7.5	[$^2H_5$]-lenvatinib (RT 6.8 min)
Dasatinib	20	16	12.8	10	8	5	2	1	[$^2H_4$]-olaparib (RT 8.8 min)
Encorafenib	50	40	32	25	20	12.5	5	2.5	[$^2H_3$]-clopidogrel (RT 9.7 min)
Enzalutamide	22500	18000	14400	11250	9000	5625	2250	1125	[$^2H_3$]-clopidogrel (RT 9.7 min)
Erlotinib	2000	1600	1280	1000	800	500	200	100	[$^2H_4$]-olaparib (RT 8.8 min)
Gefitinib	600	480	384	300	240	150	60	30	[$^2H_5$]-lenvatinib (RT 6.8 min)
Ibrutinib	150	120	96	75	60	37.5	15	7.5	[$^2H_3$]-clopidogrel (RT 9.7 min)
Idelalisib	800	640	512	400	320	200	80	40	[$^2H_4$]-olaparib (RT 8.8 min)
Imatinib	2000	1600	1280	1000	800	500	200	100	[$^2H_5$]-lenvatinib (RT 6.8 min)
Lapatinib	1600	1280	1024	800	640	400	160	80	[$^2H_3$]-clopidogrel (RT 9.7 min)
Larotrectinib	50	40	32	25	20	12.5	5	2.5	[$^2H_3$]-clopidogrel (RT 9.7 min)
Lenalidomide	30	24	19.2	15	12	7.5	3	1.5	[$^2H_5$]-lenvatinib (RT 6.8 min)
Lenvatinib	100	80	64	50	40	25	10	5	[$^2H_4$]-olaparib (RT 8.8 min)
Lorlatinib	300	240	192	150	120	75	30	15	[$^2H_5$]-lenvatinib (RT 6.8 min)
Midostaurin	2000	1600	1280	1000	800	500	200	100	[$^2H_4$]-everolimus (RT 11.4 min)
Neratinib	110	88	70.4	55	44	27.5	11	5.5	[$^2H_5$]-lenvatinib (RT 6.8 min)
Nilotinib	2400	1920	1536	1200	960	600	240	120	[$^2H_3$]-clopidogrel (RT 9.7 min)
Nintedanib	30	24	19.2	15	12	7.5	3	1.5	[$^2H_3$]-clopidogrel (RT 9.7 min)
Niraparib	1300	1040	832	650	520	325	130	65	[$^2H_5$]-lenvatinib (RT 6.8 min)
Olaparib	3000	2400	1920	1500	1200	750	300	150	[$^2H_4$]-olaparib (RT 8.8 min)
Osimertinib	350	280	224	175	140	87.5	35	17.5	[$^2H_5$]-lenvatinib (RT 6.8 min)
Palbociclib	120	96	76.8	60	48	30	12	6	[$^2H_5$]-lenvatinib (RT 6.8 min)
Panobinostat	20	16	12.8	10	8	5	2	1	[$^2H_5$]-lenvatinib (RT 6.8 min)
Pazopanib	1040	832	665.6	520	416	260	104	52	[$^2H_4$]-olaparib (RT 8.8 min)
Pomalidomide	80	64	51.2	40	32	20	8	4	[$^2H_5$]-lenvatinib (RT 6.8 min)
Ponatinib	70	56	44.8	35	28	17.5	7	3.5	[$^2H_3$]-clopidogrel (RT 9.7 min)
Regorafenib	2800	2240	1792	1400	1120	700	280	140	[$^2H_4$]-everolimus (RT 11.4 min)
Ribociclib	1500	1200	960	750	600	375	150	75	[$^2H_5$]-lenvatinib (RT 6.8 min)
Rucaparib	3500	2800	2240	1750	1400	875	350	175	[$^2H_5$]-lenvatinib (RT 6.8 min)
Ruxolitinib	25	20	16	12.5	10	6.25	2.5	1.25	[$^2H_5$]-lenvatinib (RT 6.8 min)
Sonidegib	2000	1600	1280	1000	800	500	200	100	[$^2H_4$]-everolimus (RT 11.4 min)
Sorafenib	7500	6000	4800	3750	3000	1875	750	375	[$^2H_4$]-everolimus (RT 11.4 min)
Sunitinib	100	80	64	50	40	25	10	5	[$^2H_4$]-olaparib (RT 8.8 min)
Thalidomide	1200	960	768	600	480	300	120	60	[$^2H_5$]-lenvatinib (RT 6.8 min)

Tivozanib	160	128	102.4	80	64	40	16	8	[$^2H_3$]-clopidogrel (RT 9.7 min)
Trametinib	50	40	32	25	20	12.5	5	2.5	[$^2H_4$]-everolimus (RT 11.4 min)
Trifluridine	2500	2000	1600	1250	1000	625	250	125	[$^2H_5$]-lenvatinib (RT 6.8 min)
Vandetanib	1600	1280	1024	800	640	400	160	80	[$^2H_5$]-lenvatinib (RT 6.8 min)
Vemurafenib	2000	1600	1280	1000	800	500	200	100	[$^2H_4$]-everolimus (RT 11.4 min)
Venetoclax	1100	880	704	550	440	275	110	55	[$^2H_4$]-everolimus (RT 11.4 min)
Vinorelbine	120	96	76.8	60	48	30	12	6	[$^2H_4$]-olaparib (RT 8.8 min)
Vismodegib	23000	18400	14720	11500	9200	5750	2300	1150	[$^2H_3$]-clopidogrel (RT 9.7 min)

All concentrations are given in ng/mL, STD: standard, IS: internal standard, RT: retention time, ULOV: upper limit of validation, HOV: high limit of validation, MOV: middle of validation limit , LOV: low limit of validation, LLOV: lower limit of validation.

Table S4. Oral antitumor therapy information of individual patients and measured plasma concentrations.

Patient	Oral antitumor drug	Dose [mg/day]	Dosing interval [h]	Number of blood collection	±4 h of trough level	Measured plasma concentration [ng/mL]
1	Abiraterone	1000	24	1.	yes	5.8
1	Abiraterone	1000	24	2.	yes	4.8
2	Abiraterone	1000	24	1.	yes	11.4
2.1*	Enzalutamide	160	24	1.	yes	11296.4
2.1*	Enzalutamide	160	24	2.	yes	14087.2
3	Enzalutamide	160	24	1.	yes	14395.8
3	Enzalutamide	160	24	2.	yes	17580.7
4	Abiraterone	1000	24	1.	yes	5.5
4	Abiraterone	1000	24	2.	yes	5.5
5	Cabozantinib	40/60	24	1.	yes	445.6
5	Cabozantinib	40/60	24	2.	yes	562.2
6	Abiraterone	1000	24	1.	yes	14.9
6	Abiraterone	1000	24	2.	yes	59.5
7	Cabozantinib	60	24	1.	yes	270.6
7	Cabozantinib	60	24	2.	yes	81.6
8	Enzalutamide	160	24	1.	yes	11074.7
9	Olaparib	600	12	1.	no	4442.9
9	Olaparib	600	12	2.	no	n.d.
10	Abiraterone	1000	24	1.	yes	0.4
11	Abiraterone	1000	24	1.	yes	5.9
11	Abiraterone	1000	24	2.	yes	9.7
12	Cabozantinib	40	24	1.	no	1188.9
12	Cabozantinib	40	24	2.	no	1063.2
13	Cabozantinib	60	24	1.	yes	720.3
13	Cabozantinib	60	24	2.	no	1225.4
14	Abiraterone	1000	24	1.	yes	1.8
14	Abiraterone	1000	24	2.	yes	2.0
15	Abiraterone	1000	24	1.	no	3.2
15	Abiraterone	1000	24	2.	no	8.6
16	Imatinib	400	24	1.	yes	1998.2
17	Imatinib	400	24	1.	yes	1309.6
18	Cabozantinib	20	24	1.	yes	289.4
19	Lenvatinib	14	24	1.	yes	7.8
19	Lenvatinib	14	24	2.	yes	n.d.
19.1**	Tivozanib	0.89	24	1.	yes	25.7
19.1**	Tivozanib	0.89	24	2	yes	54.6
20	Ibrutinib	420	24	1.	no	3.2
20	Ibrutinib	420	24	2.	no	4.2

21	Abiraterone	1000	24	1.	no	12.0
21	Abiraterone	1000	24	2.	no	28.2
22	Cabozantinib	20	24	1.	yes	291.0
22	Cabozantinib	20	24	2.	yes	551.4
23	Bosutinib	500	24	1.	yes	109.8
24	Osimertinib	80	24	1.	no	167.8
25	Cabozantinib	40	24	1.	no	454.1
25	Cabozantinib	40	24	2.	no	433.2
26	Cabozantinib	40	24	1.	no	870.3
26	Cabozantinib	40	24	2.	no	1217.6
27	Pazopanib	200	24	1.	yes	24300.0
27	Pazopanib	200	24	2.	yes	21520.0
28	Vandetanib	300	24	1.	no	757.7
28	Vandetanib	300	24	2.	no	621.3
29	Dabrafenib	150	12	1.	yes	212.4
29	Dabrafenib	150	12	2.	yes	460.8
30	Olaparib	600	12	1.	no	5552.4
30	Olaparib	600	12	2.	no	6053.6
31	Abiraterone	1000	24	1.	no	3.1
31	Abiraterone	1000	24	2.	no	1.4
32	Abiraterone	1000	24	1.	no	102.0
32	Abiraterone	1000	24	2.	no	166.0
33	Ibrutinib	560	24	1.	no	115.2
33	Ibrutinib	560	24	2.	no	139.5
34	Abiraterone	1000	24	1.	yes	9.9
34	Abiraterone	1000	24	2.	yes	6.3
35	Axitinib	6	12	1.	no	0.3
36	Tivozanib	1.34	24	1.	yes	13.1
36	Tivozanib	1.34	24	2.	yes	38.9
37	Cabozantinib	60	24	1.	no	700.9
37	Cabozantinib	60	24	2.	no	627.0
38	Cabozantinib	40	24	1.	yes	253.5
39	Cabozantinib	40	24	1.	no	311.3

n.d.=not detectable, * For this patient, the initial oral antitumor therapy was changed (e.g. due to tumor progression) to a second oral antitumor drug over time. The first sample was collected during the initial therapy with abiraterone. Therapy was changed to enzalutamide and two more samples were collected. ** For this patient, the initial oral antitumor therapy was changed (e.g. due to tumor progression) to a second oral antitumor drug over time. The first two samples were collected during the initial therapy with lenvatinib. Therapy was changed to tivozanib and two more samples were collected.

Table S5. Validation results of the 57 oral antitumor drugs following the FDA guideline [56].

Oral anti-cancer drug	Valida-tion standard	Intraday accuracy and precision		Interday accuracy and precision		Mean matrix effect	Mean recovery	Mean process effi-ciency	Car-ryover	Selectivity	Sensitiv-ity	Dilution in-tegrity	
		Accuracy [%]	CV [%]	Accuracy [%]	CV [%]	[%]	[%]	[%]	[%]	[%]	[%]	4-fold c_{max}	Dilu-tion
Abema-ciclib	LLOV	98.2	2.4	101.9	0.6	1131.0	93.0	1128.6	-	-	-	91.4	104.5
	2xLLOV	97.0	1.5	102.7	0.5								
	LOV	98.1	3.1	104.9	0.8								
	MOV	98.3	2.8	105.8	0.7								
	HOV	101.4	3.2	107.0	0.8								
	ULOV	95.7	2.9	101.6	0.5								
Abi-raterone	LLOV	105.5	2.4	98.2	0.8	5.0	93.4	98.1	-	-	-	109.3	103.4
	2xLLOV	98.4	3.4	97.7	1.1								
	LOV	95.6	1.4	98.5	0.7								
	MOV	99.3	3.3	101.8	0.8								
	HOV	100.3	3.6	103.8	1.1								
	ULOV	101.4	1.9	102.8	0.6								
Afatinib	LLOV	82.7	3.7	89.9	1.9	7.3	94.4	101.5	-	-	-	114.5	108.5
	2xLLOV	99.8	4.0	104.8	1.2								
	LOV	104.0	3.6	106.5	0.5								
	MOV	103.3	3.2	105.2	0.4								
	HOV	101.5	4.0	104.4	1.1								
	ULOV	98.2	1.7	98.9	0.3								
Alectinib	LLOV	92.8	2.7	92.1	0.7	6.1	94.4	100.2	-	-	-	89.4	96.8
	2xLLOV	100.9	2.0	101.9	0.5								
	LOV	102.3	2.1	104.9	0.5								
	MOV	101.9	1.8	104.3	0.4								
	HOV	101.4	2.1	104.6	0.6								
	ULOV	96.8	2.5	97.7	0.5								
Anagrelide	LLOV	114.8	9.8	102.4	1.2	7.2	94.0	100.6	-	-	-	92.7	95.2
	2xLLOV	107.8	8.0	101.9	1.5								
	LOV	97.8	9.3	98.8	2.5								
	MOV	98.1	3.2	100.6	0.6								
	HOV	97.6	3.4	101.7	0.5								
	ULOV	97.1	1.7	99.8	0.7								
Apalutam-ide	LLOV	102.6	3.7	101.9	0.9	10.5	94.0	103.8	-	-	-	109.3	104.6
	2xLLOV	99.7	2.7	101.7	0.5								
	LOV	97.8	3.3	100.9	0.6								
	MOV	100.4	2.6	102.3	0.3								
	HOV	98.8	1.5	103.2	0.5								
	ULOV	98.4	1.9	100.1	0.4								
Axitinib	LLOV	107.4	13.0	108.3	3.4	10.2	93.9	103.6	-	-	-	121.1	114.7
	2xLLOV	98.6	5.1	103.3	2.0								
	LOV	97.8	4.5	102.1	1.1								
	MOV	97.8	6.2	102.5	1.7								
	HOV	99.2	7.0	107.0	1.6								
	ULOV	99.8	4.3	105.1	1.2								
Binimetinib	LLOV	114.0	4.9	117.8	1.5	0.3	96.6	96.5	-	-	-	103.4	97.7
	2xLLOV	93.3	4.1	100.3	0.9								
	LOV	91.4	6.7	96.9	1.9								
	MOV	97.7	3.5	99.4	1.2								
	HOV	99.2	3.3	103.8	0.7								

	ULOV	103.1	2.5	105.1	0.5								
Bosutinib	LLOV	107.0	1.7	106.3	0.6	165.1	90.8	241.9	-	-	-	96.4	114.8
	2xLLOV	97.9	3.9	106.9	1.0								
	LOV	97.3	4.3	108.5	1.0								
	MOV	96.6	4.1	108.6	1.0								
	HOV	96.6	4.4	107.9	1.0								
	ULOV	95.7	3.6	105.3	1.0								
Brigatinib	LLOV	87.7	10.8	98.2	3.1	1855.0	98.0	1816.3	-	-	-	78.9	60.6
	2xLLOV	94.2	1.9	101.4	0.4								
	LOV	101.7	2.1	102.0	0.7								
	MOV	100.2	2.2	105.5	0.6								
	HOV	103.0	4.7	108.4	1.3								
	ULOV	97.6	2.2	100.6	0.7								
Cabozan-tinib	LLOV	89.4	3.4	90.0	1.0	5.8	94.6	100.1	0.4	-	-	84.4	105.0
	2xLLOV	100.4	1.9	102.7	0.3								
	LOV	104.6	3.1	107.6	1.0								
	MOV	103.5	2.2	106.2	0.5								
	HOV	100.5	2.8	104.5	0.6								
	ULOV	94.6	1.6	96.0	0.4								
Ceritinib	LLOV	108.9	1.8	106.8	0.5	8.8	92.9	101.0	-	-	-	93.9	88.3
	2xLLOV	100.9	1.9	99.6	0.3								
	LOV	97.4	2.6	97.8	0.6								
	MOV	102.0	2.4	102.1	0.6								
	HOV	101.0	3.3	104.0	0.9								
	ULOV	101.0	1.6	102.9	0.3								
Cobi-metinib	LLOV	102.6	2.9	99.7	0.5	7.1	94.7	101.5	-	-	-	105.5	94.0
	2xLLOV	98.5	3.3	99.2	1.1								
	LOV	96.1	2.6	98.7	0.5								
	MOV	98.5	2.0	101.5	0.5								
	HOV	99.2	4.2	103.2	1.1								
	ULOV	98.8	1.9	100.5	0.4								
Crizotinib	LLOV	90.6	4.2	88.6	1.1	6.8	95.2	101.6	-	-	-	69.0	108.9
	2xLLOV	105.0	2.9	107.4	0.8								
	LOV	110.3	1.7	113.4	0.7								
	MOV	103.3	2.2	105.7	0.4								
	HOV	97.6	2.3	101.5	0.4								
	ULOV	88.1	1.1	89.6	0.3								
Dabrafenib	LLOV	103.9	4.3	98.2	1.1	9.6	93.6	102.6	-	-	-	113.5	112.6
	2xLLOV	101.7	3.8	101.1	0.8								
	LOV	100.2	3.1	100.8	0.8								
	MOV	100.5	1.7	102.3	0.4								
	HOV	100.6	2.5	103.0	0.7								
	ULOV	98.4	1.4	99.8	0.1								
Dacomitinib	LLOV	86.8	2.2	90.8	1.4	5.6	92.7	97.8	-	-	-	78.1	76.1
	2xLLOV	100.2	2.5	101.7	0.8								
	LOV	105.4	2.3	106.0	0.4								
	MOV	104.6	2.6	104.7	0.5								
	HOV	103.8	2.6	105.3	0.5								
	ULOV	98.9	2.3	98.7	0.3								
Dasatinib	LLOV	119.9	8.8	118.5	1.4	14.1	89.5	100.8	-	-	-	110.2	104.3
	2xLLOV	103.5	6.9	105.1	1.0								
	LOV	98.3	6.4	97.6	1.8								
	MOV	99.3	4.8	100.0	1.5								
	HOV	98.5	6.5	103.5	1.8								
	ULOV	104.4	1.8	105.2	0.8								
	LLOV	107.9	5.5	101.3	1.6	7.4	96.8	104.1	-	-	-	130.5	122.2

	2xLLOV	103.8	2.9	103.4	1.0								
Encoraf-enib	LOV	101.0	4.3	102.7	0.5								
	MOV	104.7	3.4	103.6	0.7								
	HOV	100.7	3.4	103.6	0.8								
	ULOV	98.5	3.1	99.8	0.6								
	LLOV	95.3	3.7	97.4	0.9								
	2xLLOV	98.4	2.1	100.6	0.4								
Enzalutam-ide	LOV	99.0	2.4	101.3	0.5	8.9	94.3	102.8	0.0	-	-	87.8	100.7
	MOV	101.0	3.1	102.7	0.9								
	HOV	100.8	2.8	103.2	0.7								
	ULOV	97.0	2.4	97.8	0.6								
	LLOV	114.8	1.3	111.7	0.4								
	2xLLOV	99.8	1.8	101.7	0.6								
Erlotinib	LOV	96.0	4.5	98.9	1.2	7.6	94.5	101.7	0.1	-	-	97.3	95.4
	MOV	99.4	3.5	101.2	1.0								
	HOV	101.7	5.1	104.8	1.0								
	ULOV	101.2	2.4	103.3	0.2								
	LLOV	86.9	5.2	91.5	1.3								
	2xLLOV	96.2	2.6	101.4	0.6								
Gefitinib	LOV	102.9	1.9	106.1	0.5	5.5	94.7	100.0	-	-	-	87.0	101.5
	MOV	103.2	3.2	105.4	0.6								
	HOV	101.5	3.3	105.6	0.9								
	ULOV	96.6	0.6	98.4	0.6								
	LLOV	108.2	2.2	101.4	0.9								
	2xLLOV	100.3	3.1	100.1	1.2								
Ibrutinib	LOV	96.9	2.3	99.8	0.3	8.6	93.6	101.7	-	-	-	107.3	101.2
	MOV	99.2	2.9	101.8	0.6								
	HOV	100.0	3.3	104.0	0.8								
	ULOV	99.7	2.7	101.4	0.6								
	LLOV	116.9	1.4	117.4	0.2								
	2xLLOV	99.4	1.6	101.2	0.5								
Idelalisib	LOV	94.4	5.0	96.0	1.1	9.6	93.4	102.3	-	-	-	107.6	96.5
	MOV	97.2	3.6	99.5	0.9								
	HOV	100.4	2.7	103.9	0.6								
	ULOV	103.4	2.9	105.8	0.8								
	LLOV	99.1	2.2	103.2	1.1								
	2xLLOV	98.6	3.5	101.7	0.8								
Imatinib	LOV	100.7	4.0	104.1	0.7	153.0	93.1	235.0	-	-	-	72.7	106.7
	MOV	99.7	2.1	106.1	0.4								
	HOV	100.7	4.3	105.9	1.1								
	ULOV	95.7	3.1	99.1	0.9								
	LLOV	94.8	2.4	95.4	0.5								
	2xLLOV	103.2	2.3	105.9	0.6								
Lapatinib	LOV	105.2	1.9	108.9	0.3	6.1	93.6	99.3	4.4	-	-	91.1	100.7
	MOV	104.1	2.1	106.8	0.5								
	HOV	101.7	2.4	105.3	0.6								
	ULOV	95.5	1.1	97.6	0.2								
	LLOV	106.7	4.3	100.5	2.2								
	2xLLOV	102.4	6.1	101.8	0.9								
Larotrec-tinib	LOV	99.1	5.7	100.5	1.2	18.0	93.5	110.0	-	-	-	107.7	106.0
	MOV	96.4	4.9	102.3	1.0								
	HOV	97.2	4.9	102.8	1.3								
	ULOV	98.4	2.8	100.4	1.1								
	LLOV	85.5	11.1	88.7	2.1								
Lenalido-mide	2xLLOV	103.5	4.3	108.1	1.2	25.7	90.7	111.1	-	-	-	98.8	105.9
	LOV	105.3	3.0	107.2	1.0								

	MOV	98.5	1.6	103.0	0.9								
	HOV	99.7	4.7	105.4	1.2								
	ULOV	96.8	1.1	100.7	0.5								
Lenvatinib	LLOV	113.4	2.0	115.0	0.4	5.6	94.3	99.7	-	-	-	111.7	101.7
	2xLLOV	99.0	2.3	101.1	0.7								
	LOV	93.6	4.6	96.3	0.9								
	MOV	96.5	3.3	99.9	0.8								
	HOV	100.0	2.9	104.3	0.5								
	ULOV	104.0	2.0	105.3	0.3								
Lorlatinib	LLOV	99.0	3.0	99.8	0.7	7.4	94.5	101.5	-	-	-	104.9	98.2
	2xLLOV	98.1	2.4	99.9	0.6								
	LOV	97.6	2.2	100.2	0.5								
	MOV	100.2	1.2	101.5	0.2								
	HOV	100.2	4.0	103.5	1.1								
	ULOV	100.9	0.7	101.1	0.4								
Midostaurin	LLOV	105.1	4.9	110.0	0.9	9.0	92.8	101.2	12.7	-	-	96.6	107.6
	2xLLOV	102.7	4.8	104.7	1.4								
	LOV	100.1	3.8	103.9	1.6								
	MOV	100.6	2.5	104.1	0.9								
	HOV	102.0	4.1	106.8	1.1								
	ULOV	101.3	2.8	105.2	0.8								
Neratinib	LLOV	72.6	8.7	90.6	2.4	43.3	95.1	137.0	-	-	-	114.0	109.3
	2xLLOV	103.3	3.0	109.2	1.0								
	LOV	114.0	1.5	114.5	0.2								
	MOV	109.6	1.9	109.3	0.6								
	HOV	106.0	3.0	108.1	0.3								
	ULOV	102.9	3.6	101.3	0.5								
Nilotinib	LLOV	93.8	2.5	97.1	0.5	7.7	93.5	100.7	-	-	-	95.1	103.6
	2xLLOV	98.8	3.1	101.9	0.7								
	LOV	100.8	2.0	103.1	0.4								
	MOV	102.1	1.8	103.8	0.4								
	HOV	101.3	3.1	104.1	0.7								
	ULOV	98.0	2.1	99.8	0.3								
Nintedanib	LLOV	91.8	7.2	91.0	1.9	8.4	94.0	101.9	-	-	-	102.3	95.7
	2xLLOV	99.4	3.9	100.8	1.5								
	LOV	102.9	1.2	104.8	0.7								
	MOV	100.9	2.0	104.4	0.7								
	HOV	102.4	2.8	105.8	0.8								
	ULOV	100.7	1.5	100.9	0.3								
Niraparib	LLOV	93.2	1.9	94.5	0.4	5.9	93.1	98.5	-	-	-	100.0	102.5
	2xLLOV	96.8	1.6	99.1	0.3								
	LOV	99.2	3.1	102.2	0.8								
	MOV	99.4	2.0	102.1	0.4								
	HOV	98.7	3.2	103.7	0.8								
	ULOV	96.0	1.0	99.3	0.2								
Olaparib	LLOV	116.5	1.0	116.2	0.2	8.1	95.1	102.7	-	-	-	119.0	105.9
	2xLLOV	98.6	2.0	100.7	0.6								
	LOV	93.2	3.0	95.6	0.8								
	MOV	96.3	1.8	99.1	0.4								
	HOV	99.6	3.5	103.5	0.7								
	ULOV	102.7	2.3	104.7	0.6								
Osimertinib	LLOV	103.9	2.6	101.8	0.4	7.3	94.2	101.1	-	-	-	80.4	80.6
	2xLLOV	99.3	2.9	100.8	0.7								
	LOV	102.1	3.8	101.4	1.0								
	MOV	101.4	2.0	102.2	0.8								
	HOV	102.4	3.5	105.1	0.6								

	ULOV	100.4	2.9	99.6	0.9								
Palbociclib	LLOV	94.8	3.0	96.3	0.7	17.4	94.0	110.2	-	-	-	106.5	104.7
	2xLLOV	96.6	2.1	99.9	0.8								
	LOV	100.5	3.4	102.3	0.8								
	MOV	99.7	3.3	102.3	0.9								
	HOV	99.5	3.0	102.8	0.5								
	ULOV	98.7	1.6	99.4	0.3								
Panobinostat	LLOV	83.3	20.2	87.2	5.2	13.3	94.8	107.0	-	48.9 (LLOV), 23.4 (2XLLOV)	37.1	108.8	112.7
	2xLLOV	103.2	4.4	101.0	2.9								
	LOV	109.4	3.4	107.2	2.0								
	MOV	107.2	4.5	106.3	1.5								
	HOV	107.3	4.4	105.5	1.5								
	ULOV	101.2	3.0	100.8	1.0								
Pazopanib	LLOV	98.1	1.4	99.6	0.2	5.9	95.1	100.6	-	-	-	99.1	116.9
	2xLLOV	101.4	1.4	102.9	0.8								
	LOV	102.9	4.3	104.9	0.9								
	MOV	104.5	2.3	105.3	0.5								
	HOV	105.4	1.3	106.5	0.1								
	ULOV	102.2	1.5	102.3	0.4								
Pomalidomide	LLOV	85.2	4.9	94.5	2.2	7.2	93.9	100.5	-	-	-	103.1	101.3
	2xLLOV	95.7	6.8	98.7	2.1								
	LOV	97.8	2.6	100.8	0.6								
	MOV	98.5	2.9	101.9	0.5								
	HOV	100.6	2.7	104.8	0.6								
	ULOV	99.5	2.5	101.4	0.6								
Ponatinib	LLOV	98.7	4.8	103.5	1.1	-5.0	95.0	90.3	-	-	-	110.5	105.2
	2xLLOV	98.1	1.3	104.0	0.9								
	LOV	98.1	3.0	101.6	0.6								
	MOV	99.0	1.9	102.7	0.4								
	HOV	97.9	2.9	103.7	0.8								
	ULOV	96.0	3.2	100.0	0.9								
Regorafenib	LLOV	104.3	4.3	106.2	1.3	8.9	93.6	101.9	1.7	-	-	116.1	118.8
	2xLLOV	101.5	2.2	102.4	0.8								
	LOV	99.7	3.1	103.1	1.0								
	MOV	100.9	2.6	103.4	0.5								
	HOV	101.3	4.7	106.2	1.1								
	ULOV	100.7	2.4	103.9	0.5								
Ribociclib	LLOV	89.7	2.3	87.7	0.8	12.0	93.8	104.8	-	-	-	64.6	104.1
	2xLLOV	102.8	1.8	106.7	0.9								
	LOV	107.6	1.3	113.5	0.7								
	MOV	98.4	3.4	105.4	0.7								
	HOV	94.1	3.4	102.4	0.6								
	ULOV	86.3	1.0	92.0	0.6								
Rucaparib	LLOV	99.5	2.1	98.3	0.6	6.8	94.1	100.5	-	-	-	106.6	116.1
	2xLLOV	94.1	1.8	95.7	0.4								
	LOV	100.8	1.3	102.5	0.2								
	MOV	104.1	2.3	105.8	0.5								
	HOV	104.8	1.0	107.0	0.4								
	ULOV	101.7	1.3	100.6	0.5								
Ruxolitinib	LLOV	101.7	5.4	100.1	1.2	6.0	94.7	100.4	-	-	-	102.8	101.7
	2xLLOV	99.8	1.5	102.1	0.7								
	LOV	100.3	2.7	101.6	0.5								
	MOV	100.9	1.3	102.2	0.6								
	HOV	101.2	3.9	103.8	1.1								
	ULOV	100.4	1.6	100.5	0.4								
Sonidegib	LLOV	102.1	2.2	105.0	0.4	8.5	93.9	102.0	-	-	-	109.3	110.1

	2xLLOV	101.9	2.3	103.4	0.7								
	LOV	102.1	1.5	103.9	1.3								
	MOV	101.3	1.9	103.8	0.9								
	HOV	101.3	3.5	106.5	1.0								
	ULOV	101.8	1.9	105.3	0.6								
Sorafenib	LLOV	102.1	2.1	105.3	0.5	8.5	94.2	102.2	2.7	-	-	105.8	114.3
	2xLLOV	102.7	2.8	103.0	0.6								
	LOV	101.9	2.2	103.8	1.3								
	MOV	102.0	2.0	103.8	0.8								
	HOV	101.7	5.0	106.7	1.2								
	ULOV	101.5	1.7	104.8	0.9								
Sunitinib	LLOV	117.7	1.9	113.3	0.5	13.2	93.8	106.2	-	-	-	115.0	112.5
	2xLLOV	102.3	1.2	102.3	0.6								
	LOV	100.4	4.2	99.5	1.1								
	MOV	102.9	0.8	103.6	0.5								
	HOV	105.5	1.4	107.6	0.8								
	ULOV	109.4	1.0	108.0	0.7								
Thalido-mide	LLOV	117.2	5.3	107.8	1.0	11.7	93.2	104.2	-	-	-	102.5	89.7
	2xLLOV	111.6	5.4	108.2	0.9								
	LOV	111.9	2.2	110.2	0.7								
	MOV	108.7	5.7	110.5	1.5								
	HOV	110.7	1.3	112.8	1.4								
	ULOV	107.9	6.6	106.6	1.8								
Tivozanib	LLOV	97.3	2.9	99.1	0.5	5.8	94.2	99.8	-	-	-	103.4	101.2
	2xLLOV	99.1	2.2	101.6	0.5								
	LOV	99.3	1.3	102.1	0.2								
	MOV	101.8	2.4	103.1	0.6								
	HOV	100.7	3.8	103.7	0.9								
	ULOV	98.4	2.4	99.5	0.6								
Trametinib	LLOV	103.2	4.3	99.5	2.2	7.7	94.7	102.0	-	-	-	112.5	110.0
	2xLLOV	104.1	5.7	104.9	1.1								
	LOV	105.2	5.3	108.3	0.8								
	MOV	100.3	2.2	106.0	1.0								
	HOV	98.8	3.7	105.8	0.8								
	ULOV	93.2	1.5	101.5	0.7								
Tri-fluridine	LLOV	88.3	4.2	81.7	0.4	7.0	96.0	102.7	-	-	-	118.3	151.6
	2xLLOV	107.3	5.6	108.6	1.1								
	LOV	105.8	1.3	110.6	0.5								
	MOV	103.1	2.9	106.0	0.5								
	HOV	100.4	3.8	106.1	1.1								
	ULOV	97.0	1.1	100.4	0.5								
Vandetanib	LLOV	86.8	3.5	87.0	0.9	5.2	94.6	99.4	-	-	-	74.6	104.7
	2xLLOV	100.5	1.7	102.8	0.5								
	LOV	107.9	1.2	109.4	0.4								
	MOV	103.7	1.6	105.5	0.3								
	HOV	100.1	3.4	103.4	0.8								
	ULOV	92.5	0.9	93.4	0.5								
Vemuraf-enib	LLOV	105.9	4.6	104.6	0.4	8.9	92.1	100.3	0.5	-	-	113.5	115.0
	2xLLOV	103.9	3.6	103.6	0.7								
	LOV	102.6	2.7	105.1	1.4								
	MOV	101.1	1.2	104.5	1.2								
	HOV	101.1	4.7	106.4	1.0								
	ULOV	103.5	2.0	105.9	0.8								
Venetoclax	LLOV	108.5	5.7	106.8	1.1	11.8	89.0	99.4	-	-	-	126.7	114.9
	2xLLOV	101.5	4.4	102.0	1.7								
	LOV	99.6	3.9	102.1	1.4								

	MOV	103.3	2.4	103.1	1.0								
	HOV	103.5	5.4	105.4	1.5								
	ULOV	104.9	3.0	106.1	0.6								
Vinorelbine	LLOV	103.3	8.0	108.7	1.6	12.3	97.9	110.1	-	16.4	-	94.6	85.4
	2xLLOV	94.8	6.1	98.6	1.5								
	LOV	92.9	2.9	97.3	0.7								
	MOV	98.6	4.1	100.9	0.4								
	HOV	101.2	2.8	104.4	0.9								
	ULOV	100.1	4.0	104.2	0.7								
Vismodegib	LLOV	86.7	3.5	87.7	1.0	9.4	94.9	103.8	-	-	-	76.6	107.4
	2xLLOV	102.1	2.4	105.2	0.5								
	LOV	107.4	3.3	108.9	1.0								
	MOV	105.1	2.7	105.7	0.7								
	HOV	101.7	2.4	104.6	0.7								
	ULOV	96.0	1.3	96.7	0.3								

ULOV: upper limit of validation, HOV: high limit of validation, MOV: middle of validation limit, LOV: low limit of validation, LLOV: lower limit of validation.

Table S6. Validation results of stability tests.

Oral anticancer drug	Validation standard	Freeze-Thaw		Short-time stability (24h, RT, with daylight)		Short-time stability (24h, RT, without daylight)		Short-time stability (24h, 4°C, without daylight)		Short-time stability (1h, 56°C)		Autosampler (24h, 4°C)		Autosampler (72h, 4°C)	
		Accuracy [%]	CV [%]	Accuracy [%]	CV [%]	Accuracy [%]	CV [%]	Accuracy [%]	CV [%]	Accuracy [%]	CV [%]	Accuracy [%]	CV [%]	Accuracy [%]	CV [%]
Abemaciclib	LLOV	90.4	4.0	90.1	2.5	88.2	3.0	93.7	2.0	27.4	6.8	89.1	2.8	86.1	1.4
	2xLLOV	96.4	1.3	88.7	1.3	87.4	2.6	102.9	2.5	27.9	9.3	99.5	2.6	95.3	3.4
	MOV	102.7	3.8	90.3	3.8	91.7	3.3	104.5	2.0	34.3	2.5	100.7	6.1	96.7	3.1
	ULOV	105.3	0.8	88.4	2.4	87.5	3.3	94.4	1.6	34.9	5.7	102.6	1.3	100.1	1.7
Abiraterone	LLOV	105.9	6.8	105.6	4.7	105.2	6.0	104.6	5.3	49.9	4.0	90.8	1.9	94.9	1.4
	2xLLOV	104.5	3.0	101.7	2.3	101.5	2.4	96.9	2.3	46.5	3.6	98.9	1.8	97.9	3.0
	MOV	101.5	2.2	103.1	1.3	101.1	1.5	97.4	2.5	40.7	0.8	104.6	1.2	101.6	1.6
	ULOV	100.2	1.7	103.3	0.6	102.0	1.5	95.4	1.9	34.9	4.7	100.2	0.4	104.1	1.3
Afatinib	LLOV	93.5	9.5	64.3	6.7	69.9	7.1	85.9	5.4	7.2	11.4	91.7	6.6	83.0	7.2
	2xLLOV	100.0	4.8	80.8	12.2	92.5	8.6	85.5	8.4	30.4	1.3	103.2	5.0	99.3	4.0
	MOV	96.1	0.8	89.4	4.6	91.4	2.6	97.9	3.0	36.2	0.6	102.6	0.5	103.4	3.6
	ULOV	95.0	1.1	77.8	2.3	82.7	1.6	92.8	2.2	33.2	3.9	98.7	1.3	99.3	2.3
Alectinib	LLOV	91.2	3.0	90.4	1.5	92.9	0.6	95.8	1.2	22.6	6.6	91.8	0.5	93.7	4.3
	2xLLOV	100.0	0.8	105.4	1.6	106.9	1.0	101.4	0.8	44.8	3.2	99.6	1.3	102.6	1.6
	MOV	101.2	1.8	107.0	2.2	111.4	1.6	100.7	0.7	50.3	1.2	104.1	1.6	104.1	1.3
	ULOV	96.5	0.5	104.6	0.4	107.8	0.8	95.5	0.4	42.9	5.3	99.3	0.6	100.6	1.7
Anagrelide	LLOV	92.8	10.4	78.3	8.4	67.4	6.1	90.4	6.1	55.6	4.9	111.7	4.1	110.5	11.7
	2xLLOV	96.5	3.1	84.8	7.0	84.4	8.7	104.2	5.0	66.3	6.2	113.2	7.9	106.1	4.3
	MOV	93.8	3.4	96.0	1.7	88.7	1.1	96.9	4.2	52.9	3.3	104.6	1.4	96.8	2.3
	ULOV	99.6	1.5	98.5	2.5	94.1	3.2	93.5	1.3	46.2	5.2	101.8	1.5	98.2	0.7
Apalutamide	LLOV	93.8	2.2	72.0	2.3	75.7	2.6	96.9	0.2	37.6	5.0	97.2	1.2	102.8	0.6
	2xLLOV	95.6	0.3	76.8	3.6	80.8	1.5	93.0	1.3	46.5	3.1	102.1	1.8	105.1	3.0
	MOV	94.8	2.4	75.7	2.2	75.2	0.8	84.3	1.6	41.2	0.8	106.8	1.2	106.0	3.4
	ULOV	95.7	1.7	63.9	1.7	63.8	1.6	84.2	1.6	29.0	3.6	102.6	2.3	106.5	2.7
Axitinib	LLOV	102.1	10.4	108.7	26.7	106.1	3.7	129.6	4.5	71.1	8.7	105.9	8.8	97.7	5.0
	2xLLOV	108.3	9.0	107.5	11.2	86.7	7.1	103.6	3.6	67.7	2.7	101.2	7.6	104.9	2.3
	MOV	105.4	2.7	116.9	6.1	99.3	1.4	100.6	1.4	54.6	2.3	102.7	0.6	100.0	3.1
	ULOV	104.0	1.6	123.3	7.6	101.6	6.5	97.2	1.2	46.2	4.6	100.4	1.2	99.1	0.9
Binimetinib	LLOV	93.8	4.8	111.6	7.7	106.0	5.2	117.1	10.4	68.1	7.5	103.1	2.8	98.1	3.0
	2xLLOV	92.6	4.9	89.3	3.4	86.4	3.3	105.5	7.3	55.9	5.4	104.7	1.9	101.6	3.1
	MOV	100.1	2.1	86.5	1.4	89.2	1.6	94.8	2.0	42.6	1.8	105.0	3.0	95.8	2.4

	ULOV	105.2	2.0	101.8	1.7	97.3	3.0	96.8	1.4	36.7	5.0	102.1	2.0	102.4	1.4
Bosutinib	LLOV	102.2	12.3	77.5	1.9	75.8	2.6	75.7	8.7	28.8	6.6	92.5	2.8	89.8	5.3
	2xLLOV	89.9	6.1	84.6	0.7	79.7	0.4	94.4	3.8	40.5	1.4	99.6	4.3	92.9	3.1
	MOV	92.6	3.5	91.2	1.5	86.1	2.9	99.6	1.7	43.1	4.1	108.1	3.8	101.8	6.7
	ULOV	94.6	0.5	93.1	4.9	93.6	0.5	88.4	3.7	38.9	4.9	100.3	5.4	95.2	4.8
Brigatinib	LLOV	111.4	3.8	55.6	2.1	55.1	2.6	95.0	4.9	7.8	0.0	90.7	2.0	85.3	0.3
	2xLLOV	98.7	1.1	76.4	1.2	74.1	2.5	102.8	3.0	18.2	9.4	96.4	2.3	91.9	1.0
	MOV	95.5	3.6	85.3	12.3	93.4	3.1	104.2	0.9	38.5	2.1	104.4	2.4	95.5	2.4
	ULOV	97.9	1.0	91.3	2.5	90.3	1.8	93.7	0.2	35.7	4.4	96.1	2.7	96.6	1.6
Cabozan-tinib	LLOV	90.2	2.7	91.8	1.4	90.7	1.2	98.5	2.5	30.2	5.3	92.0	1.3	94.0	2.2
	2xLLOV	99.6	0.8	103.6	2.5	101.0	1.7	101.8	0.5	49.1	3.5	100.8	1.6	103.4	1.6
	MOV	102.2	1.6	104.9	1.3	108.0	0.6	102.2	1.2	51.8	0.5	105.6	0.9	103.5	2.0
	ULOV	95.0	0.4	100.6	0.4	99.9	1.4	96.1	1.5	44.1	4.5	98.7	1.3	101.3	2.7
Ceritinib	LLOV	99.9	2.5	70.9	1.2	77.7	1.0	102.1	1.4	42.5	3.3	97.1	0.5	98.1	0.9
	2xLLOV	96.9	0.8	66.9	2.2	72.2	0.4	95.5	0.2	39.7	2.2	95.1	3.1	100.1	2.7
	MOV	97.6	1.7	84.1	2.6	92.2	0.9	93.9	3.1	34.6	0.9	100.8	1.8	103.5	1.8
	ULOV	97.5	1.0	91.8	3.2	96.2	1.2	92.7	0.4	32.0	4.2	101.3	0.8	106.5	1.5
Cobi-metinib	LLOV	98.3	1.7	98.6	1.9	99.8	2.7	99.9	1.9	42.7	5.6	94.6	1.5	93.0	4.8
	2xLLOV	98.6	1.3	98.3	1.9	98.8	1.4	100.9	1.6	51.4	2.6	100.3	2.4	100.8	2.4
	MOV	97.3	1.3	102.4	2.6	103.8	2.2	102.5	0.3	47.6	1.5	105.2	2.7	103.1	2.0
	ULOV	96.3	1.4	103.8	1.7	100.3	0.5	97.5	1.4	41.3	4.1	102.5	2.5	107.2	2.7
Crizotinib	LLOV	72.2	3.5	62.9	0.6	71.2	1.3	84.5	0.5	21.3	8.5	88.1	1.8	86.1	0.6
	2xLLOV	94.8	0.9	85.9	2.4	92.4	2.6	96.1	1.4	49.5	1.9	105.2	1.6	103.5	0.9
	MOV	102.4	2.7	95.3	0.9	99.8	1.0	101.0	1.9	54.6	2.0	108.4	2.0	103.8	2.3
	ULOV	92.4	2.0	85.2	3.0	89.5	1.9	85.8	1.8	45.2	4.1	96.5	0.5	94.7	0.8
Dabrafenib	LLOV	93.9	4.2	97.0	1.2	108.2	8.0	112.7	4.9	42.8	8.8	93.5	4.2	93.1	2.2
	2xLLOV	97.8	1.4	98.0	4.9	107.6	3.7	99.6	2.1	46.2	0.4	97.9	3.0	99.3	1.7
	MOV	99.0	2.3	97.7	2.2	99.3	3.6	100.0	2.2	42.4	1.3	102.1	1.0	99.8	3.3
	ULOV	97.6	1.5	100.2	0.7	99.7	2.0	94.0	2.1	35.6	5.0	100.7	1.5	101.4	1.3
Dacomitini b	LLOV	89.8	2.6	74.6	1.7	72.7	4.9	90.8	1.7	10.3	5.0	93.3	1.6	92.3	4.1
	2xLLOV	92.5	2.1	86.7	1.7	88.5	1.1	94.9	2.8	30.6	4.4	102.5	0.2	99.5	2.8
	MOV	95.9	4.4	89.8	1.5	91.1	1.5	97.9	2.2	37.3	0.8	104.5	1.5	100.5	2.8
	ULOV	96.3	3.4	85.6	2.8	89.1	1.8	92.4	1.8	33.3	3.7	98.4	1.2	98.0	1.7
Dasatinib	LLOV	108.8	5.7	90.7	9.9	115.9	8.7	124.4	5.4	65.9	6.3	116.5	15.5	114.9	7.31
	2xLLOV	99.1	4.6	90.1	3.6	98.3	9.3	102.2	14.1	47.7	11.9	102.9	4.9	106.3	4.81
	MOV	99.1	1.0	91.7	0.6	90.0	2.1	100.1	1.5	46.4	2.5	102.8	1.6	99.0	3.27
	ULOV	102.2	2.8	99.4	0.2	93.1	0.9	96.7	4.0	38.9	3.4	102.6	0.6	101.0	1.29
Encorafenib	LLOV	90.4	2.3	108.1	11.6	104.0	15.7	110.1	7.4	n.a.	n.a.	87.7	7.3	98.2	6.5
	2xLLOV	94.9	3.3	109.5	1.6	112.9	1.8	106.5	6.8	54.9	5.9	102.4	7.4	102.6	5.4
	MOV	101.4	1.0	105.9	1.3	109.8	3.2	102.1	1.9	54.0	1.9	112.5	1.3	110.5	6.9
	ULOV	98.7	0.7	108.8	2.6	108.3	1.1	98.5	1.5	44.5	6.4	100.5	1.3	102.4	1.8
Enzalutam-ide	LLOV	87.6	1.7	100.4	1.5	103.0	1.0	102.0	1.1	50.0	5.9	89.9	3.5	91.3	1.7
	2xLLOV	97.8	1.4	106.1	0.2	108.3	1.2	103.3	1.6	59.4	4.4	100.5	2.8	102.2	0.9
	MOV	100.9	1.0	108.7	0.3	107.8	0.3	98.9	2.8	58.9	1.1	108.0	1.7	105.7	3.0
	ULOV	96.1	1.0	103.4	1.2	103.8	0.8	95.8	1.0	50.9	4.8	101.7	1.8	103.3	3.1
Erlotinib	LLOV	113.3	2.1	105.0	0.9	107.4	0.6	110.4	1.0	69.2	5.3	104.7	4.6	103.9	4.8
	2xLLOV	100.9	0.6	93.7	1.6	95.3	1.2	100.7	1.2	61.0	2.1	102.3	4.0	98.1	2.1
	MOV	100.4	1.1	94.9	0.8	97.9	1.2	98.0	2.0	52.1	1.1	103.3	0.9	98.3	3.1
	ULOV	102.0	1.2	105.1	0.9	101.0	3.0	98.7	1.1	45.8	4.9	103.4	2.7	102.9	2.5
Gefitinib	LLOV	92.4	1.5	83.0	3.3	83.7	1.1	90.9	0.8	33.3	5.8	91.6	3.1	90.7	1.8
	2xLLOV	97.6	0.4	91.9	2.3	92.1	2.3	97.4	0.6	46.0	2.9	102.3	1.8	98.5	0.7
	MOV	97.8	2.3	96.2	0.5	93.8	0.2	103.4	3.0	49.1	1.0	105.6	2.6	102.2	1.8
	ULOV	97.1	1.6	88.3	2.7	87.7	1.5	94.9	2.2	42.1	4.6	96.7	1.0	97.0	1.5
Ibrutinib	LLOV	100.2	5.2	76.2	3.2	75.3	0.6	92.9	5.2	38.7	0.0	97.6	3.4	95.8	1.2
	2xLLOV	100.8	1.5	72.6	2.6	72.1	0.6	94.5	1.2	29.6	2.8	101.4	2.2	100.0	1.2
	MOV	93.3	1.3	66.7	1.4	67.2	0.4	99.7	2.7	21.5	0.4	103.0	0.2	103.7	1.8

	ULOV	95.6	1.4	64.5	0.1	63.7	1.1	95.9	2.1	16.3	2.1	100.0	2.4	102.6	1.3
Idelalisib	LLOV	110.6	1.7	107.3	2.3	108.7	0.7	108.5	2.0	75.1	5.1	109.4	4.1	109.4	4.3
	2xLLOV	98.2	0.9	90.0	0.3	89.8	0.6	100.1	1.6	59.8	2.4	103.2	2.8	98.6	2.8
	MOV	96.5	1.1	89.7	0.3	89.6	0.4	97.8	1.6	46.6	1.7	102.6	0.6	97.1	3.1
	ULOV	101.5	0.9	100.9	1.2	95.0	2.8	98.7	2.4	41.4	5.4	103.9	1.1	101.4	1.1
Imatinib	LLOV	100.6	5.3	83.7	3.2	83.8	1.1	81.3	9.5	31.4	6.6	102.3	7.0	103.7	3.8
	2xLLOV	98.5	2.6	86.0	1.6	82.7	3.3	105.2	2.1	37.7	1.8	115.2	2.5	116.1	3.7
	MOV	96.7	4.1	93.6	1.8	88.6	1.8	102.2	3.3	41.9	1.7	108.3	2.5	101.7	2.1
	ULOV	96.3	0.6	85.6	2.0	82.9	3.3	94.2	2.5	36.4	4.4	95.3	1.1	95.2	0.8
Lapatinib	LLOV	86.5	2.9	5.2	2.5	14.4	1.4	92.5	1.7	-24.8	4.2	92.7	3.5	94.4	3.6
	2xLLOV	96.0	1.9	46.9	3.7	53.0	2.1	96.4	0.7	19.2	3.8	99.4	2.8	102.1	0.4
	MOV	102.3	1.7	93.5	4.1	109.4	2.7	97.9	1.9	46.1	1.3	103.2	2.3	101.5	2.2
	ULOV	94.7	1.4	100.7	3.9	112.4	1.2	94.3	0.5	42.5	4.7	99.7	1.1	98.8	1.7
Larotrec-tinib	LLOV	97.9	12.0	115.6	1.4	100.1	6.8	107.8	2.9	44.4	11.8	79.4	2.9	75.5	8.8
	2xLLOV	100.6	2.2	109.3	3.1	102.4	6.1	104.3	0.8	58.9	2.7	87.6	6.4	100.3	3.0
	MOV	100.3	3.8	106.3	0.2	110.8	2.0	99.9	4.0	58.2	2.3	104.6	2.0	101.2	3.9
	ULOV	102.3	1.6	105.3	3.1	104.5	2.7	98.3	1.6	50.9	6.7	102.3	0.5	104.4	2.3
Lenalido-mide	LLOV	91.0	1.7	81.5	0.7	n.a.	n.a.	83.0	3.1	n.a.	n.a.	80.7	10.4	95.6	3.7
	2xLLOV	104.4	7.7	54.9	4.6	49.6	4.7	107.4	2.3	39.6	0.1	117.5	4.8	109.8	5.5
	MOV	97.3	1.5	58.7	2.3	54.1	2.1	97.8	1.9	20.6	0.5	107.0	2.5	100.0	2.2
	ULOV	96.8	2.3	51.9	1.6	50.9	0.7	90.4	1.1	18.5	2.5	100.8	0.9	98.9	0.9
Lenvatinib	LLOV	111.6	0.2	106.4	2.3	110.0	1.6	114.0	3.2	62.9	4.2	110.0	6.1	103.7	4.4
	2xLLOV	102.5	1.6	96.8	1.6	94.6	1.2	103.4	4.7	56.5	0.8	102.4	5.1	97.9	1.0
	MOV	98.4	0.8	93.9	1.7	94.3	1.6	95.9	1.3	46.6	1.2	100.6	1.9	98.6	2.6
	ULOV	103.0	2.6	102.7	0.9	97.9	1.3	100.4	4.3	40.6	4.3	102.6	1.6	102.7	3.6
Lorlatinib	LLOV	96.0	0.7	96.4	1.3	97.8	0.9	98.8	1.0	54.3	7.7	96.7	1.5	94.8	2.4
	2xLLOV	95.0	0.4	98.9	0.8	99.6	1.3	99.8	1.7	60.0	3.1	101.6	0.7	99.0	1.4
	MOV	95.2	3.6	100.2	0.5	99.2	0.3	101.8	1.6	57.8	1.1	102.0	1.3	100.8	0.5
	ULOV	97.6	1.9	100.4	1.9	99.9	1.3	97.0	0.8	50.7	5.8	99.7	0.4	99.2	0.5
Midostau-rin	LLOV	100.6	1.3	89.5	1.8	85.7	5.2	90.3	7.3	39.0	3.9	109.7	5.8	108.2	2.1
	2xLLOV	95.9	4.8	91.7	3.5	88.1	2.3	93.5	5.2	45.3	3.1	103.3	1.1	103.0	3.9
	MOV	94.6	4.6	88.3	2.6	91.0	2.0	103.7	2.8	41.7	1.9	101.4	1.8	103.7	3.2
	ULOV	97.4	4.4	89.5	2.3	88.4	2.0	98.8	4.7	37.7	5.5	102.8	2.6	97.9	2.3
Neratinib	LLOV	64.7	7.4	-37.5	12.0	-39.1	11.7	65.9	7.9	-88.6	0.7	75.2	3.3	73.8	1.9
	2xLLOV	82.2	6.1	23.2	5.6	25.4	5.9	79.9	3.4	-7.9	3.2	95.7	2.9	100.1	3.1
	MOV	88.9	2.7	37.9	1.6	39.2	1.4	89.7	3.1	26.0	2.7	103.1	3.7	100.5	2.6
	ULOV	87.2	0.5	28.5	1.9	31.5	1.4	81.3	2.0	21.9	3.4	93.3	2.3	96.0	0.6
Nilotinib	LLOV	92.8	2.5	94.9	2.6	96.7	1.5	96.7	1.6	28.5	5.4	88.1	0.3	92.6	2.4
	2xLLOV	98.5	2.4	107.8	2.9	108.6	2.0	101.2	0.5	47.8	3.6	97.8	3.1	101.2	0.3
	MOV	100.9	1.4	110.2	1.4	114.5	0.2	100.3	2.6	52.5	0.7	102.7	1.1	104.0	2.0
	ULOV	96.0	1.4	108.0	1.9	110.3	0.6	97.1	0.1	44.6	5.2	100.3	1.8	102.0	1.9
Nintedanib	LLOV	93.2	4.7	99.4	13.3	96.1	3.5	96.7	2.0	56.0	0.0	91.3	4.5	99.8	6.3
	2xLLOV	103.0	2.9	102.5	6.8	105.4	4.1	103.5	3.7	48.8	4.0	104.2	1.4	104.4	3.6
	MOV	96.9	1.5	104.3	1.4	103.0	1.8	100.1	1.6	51.2	0.6	102.7	3.1	104.6	1.7
	ULOV	95.8	2.4	105.8	2.0	102.1	2.1	88.8	3.9	43.9	5.2	102.7	0.6	101.4	0.4
Niraparib	LLOV	98.0	0.7	92.3	1.0	91.9	1.5	93.8	1.7	46.8	5.7	96.8	1.1	96.3	2.7
	2xLLOV	96.8	1.5	95.2	1.4	93.8	0.3	96.9	0.4	53.4	1.9	103.0	0.9	99.6	0.5
	MOV	96.5	2.6	97.6	0.5	96.5	1.1	100.2	0.8	51.1	2.3	104.6	0.5	100.5	1.6
	ULOV	97.1	1.6	95.8	2.9	94.0	1.1	92.7	2.2	43.8	4.8	100.8	1.0	100.1	1.3
Olaparib	LLOV	113.4	0.7	108.3	0.7	110.6	0.2	110.7	0.2	80.6	5.8	111.6	4.2	111.2	3.0
	2xLLOV	99.5	0.8	91.3	0.5	90.8	0.3	101.8	0.7	64.1	1.9	103.8	1.8	100.5	2.1
	MOV	96.6	1.2	89.4	1.1	88.9	1.3	98.6	1.7	49.4	1.2	100.5	0.9	98.0	2.4
	ULOV	100.5	1.3	99.0	0.8	94.2	2.1	99.7	1.9	42.9	5.5	103.8	1.2	100.5	0.9
Osimertinib	LLOV	95.6	2.5	62.0	1.6	60.5	0.5	85.8	1.7	28.7	3.1	90.3	1.3	91.2	0.9
	2xLLOV	93.7	0.8	61.4	1.4	61.7	0.7	95.8	2.9	29.9	2.0	104.2	4.5	99.1	1.0
	MOV	96.2	2.6	75.7	1.5	72.0	2.0	96.3	0.9	30.1	1.2	103.8	1.5	100.3	3.3

	ULOV	92.7	1.4	67.4	2.0	63.9	2.0	86.7	1.4	26.0	2.7	98.4	1.8	99.1	2.3
Palbociclib	LLOV	98.8	2.4	82.9	3.1	85.8	1.3	83.5	1.8	47.3	5.4	99.4	1.2	97.9	0.9
	2xLLOV	97.7	1.1	84.1	1.4	87.0	1.1	96.8	3.1	50.8	1.6	99.9	1.0	100.1	1.4
	MOV	93.4	1.7	89.0	0.8	92.3	0.4	96.0	2.9	46.3	1.4	101.0	1.2	96.9	2.2
	ULOV	96.5	1.4	92.7	3.9	96.9	2.3	92.8	1.8	41.0	4.3	103.9	1.4	102.9	1.3
Panobino-stat	LLOV	104.3	12.9	-0.2	12.5	52.8	39.2	66.1	17.2	1.9	17.9	83.8	7.5	73.7	10.6
	2xLLOV	107.5	9.4	53.1	14.8	45.4	14.1	81.5	15.2	27.6	2.1	100.3	4.6	95.4	7.9
	MOV	98.8	2.7	92.2	2.2	96.2	3.3	93.6	1.6	48.8	2.4	102.4	2.6	101.0	1.6
	ULOV	102.3	4.1	96.0	4.5	100.4	1.6	89.2	1.7	46.3	6.6	95.4	8.3	101.1	1.6
Pazopanib	LLOV	102.7	1.2	100.2	2.9	105.8	0.7	104.1	0.1	53.4	6.9	101.3	3.7	99.0	3.3
	2xLLOV	103.5	0.7	103.3	1.9	104.3	1.7	101.2	1.8	62.0	3.0	103.7	3.6	99.4	2.1
	MOV	103.9	0.7	99.1	1.6	101.2	1.3	97.8	0.3	55.7	1.2	101.7	0.5	97.9	2.1
	ULOV	100.5	2.2	102.6	1.2	99.1	2.1	97.1	2.5	47.4	4.2	99.8	1.6	99.0	3.4
Pomalido-mide	LLOV	74.4	3.7	42.4	4.3	42.6	5.0	92.5	4.0	38.4	0.0	104.9	1.5	96.5	1.8
	2xLLOV	90.9	4.4	51.2	3.4	51.3	3.9	94.1	4.6	21.3	0.0	104.1	5.1	103.5	2.6
	MOV	90.1	2.8	51.3	2.7	51.2	1.4	96.7	3.2	20.6	1.7	104.5	2.2	97.7	2.9
	ULOV	92.4	3.3	43.6	2.2	43.8	1.1	88.6	2.6	16.9	2.1	100.5	2.0	97.9	2.2
Ponatinib	LLOV	101.5	1.8	89.6	4.9	91.7	0.6	95.7	3.5	30.7	4.6	104.7	8.4	104.7	4.1
	2xLLOV	101.8	4.9	98.7	2.3	98.1	0.9	96.6	3.0	48.3	3.6	106.1	2.9	106.1	0.9
	MOV	99.3	1.4	99.8	0.3	102.6	0.8	100.1	0.3	50.8	0.9	106.1	2.4	102.3	2.2
	ULOV	100.2	1.1	97.8	0.8	98.9	2.3	93.3	1.9	43.1	3.9	101.9	1.5	102.0	1.4
Regorafenib	LLOV	101.6	1.3	88.6	3.8	80.3	3.9	83.3	6.7	32.3	3.2	107.3	5.4	102.1	4.0
	2xLLOV	97.1	2.8	89.4	4.3	83.8	4.0	95.0	2.9	41.3	4.0	101.6	1.1	101.1	3.3
	MOV	96.2	4.7	90.2	2.1	90.8	1.3	104.6	2.7	39.7	0.9	101.4	2.3	104.4	2.1
	ULOV	98.6	2.0	89.0	1.5	87.8	1.0	101.4	2.7	35.4	5.5	104.2	2.0	99.3	5.0
Ribociclib	LLOV	86.3	2.6	79.3	1.5	81.3	1.7	87.3	0.8	30.6	7.3	84.0	0.6	82.1	2.1
	2xLLOV	103.9	0.6	94.2	1.1	92.7	0.4	98.0	3.6	49.7	1.7	103.0	2.9	102.5	1.7
	MOV	101.6	1.4	95.0	0.9	93.5	0.6	98.8	2.8	50.5	0.9	104.1	1.6	101.7	1.6
	ULOV	92.4	2.1	84.8	2.3	83.3	1.3	84.9	1.2	41.5	4.1	89.8	1.2	92.1	0.5
Rucaparib	LLOV	104.4	0.1	100.0	1.7	99.7	2.0	106.8	0.8	60.9	4.6	104.5	0.7	104.1	2.2
	2xLLOV	93.7	0.8	94.3	1.2	95.8	1.8	94.0	2.2	56.7	2.0	98.1	1.8	96.4	0.8
	MOV	97.8	1.6	100.5	1.1	102.1	1.4	102.0	0.5	52.2	1.9	104.6	2.3	103.8	1.4
	ULOV	95.5	1.8	98.9	2.2	99.1	2.1	96.9	2.2	46.1	5.0	100.1	0.0	102.4	0.1
Ruxolitinib	LLOV	92.5	2.4	100.4	11.9	105.0	1.1	102.2	1.2	63.3	4.7	93.9	0.8	94.4	2.3
	2xLLOV	94.7	1.2	101.6	2.0	102.0	0.6	97.6	3.1	63.9	2.6	100.5	2.0	97.7	2.1
	MOV	96.2	2.0	100.6	1.3	102.2	1.5	100.3	2.2	60.9	1.4	102.4	0.8	99.7	1.4
	ULOV	97.2	1.9	102.3	1.8	101.3	1.4	95.9	2.8	53.0	5.2	100.1	0.5	99.7	1.5
Sonidegib	LLOV	103.7	2.2	96.2	1.9	86.1	4.3	96.4	4.3	38.1	3.1	109.3	6.7	100.0	0.9
	2xLLOV	97.9	3.6	95.7	3.3	91.1	4.3	99.1	4.1	47.0	4.1	101.5	2.6	101.9	4.2
	MOV	96.5	4.3	92.2	1.9	94.7	2.2	98.4	3.6	44.9	0.8	99.4	1.8	101.1	3.1
	ULOV	99.0	2.1	92.9	2.1	91.4	2.5	96.7	1.9	40.1	5.4	101.3	3.1	98.1	4.5
Sorafenib	LLOV	103.3	3.1	83.4	1.1	72.1	3.4	96.0	4.0	28.2	3.4	112.0	7.0	104.9	0.3
	2xLLOV	97.6	4.1	85.6	2.3	81.9	1.6	97.0	3.8	38.6	4.0	103.1	1.2	104.1	5.0
	MOV	96.9	4.0	89.2	2.1	91.9	1.9	98.2	4.8	38.4	0.4	101.0	1.1	103.5	3.6
	ULOV	98.7	2.5	91.0	1.5	89.2	1.3	92.5	3.6	34.6	4.7	101.7	3.9	98.3	2.8
Sunitinib	LLOV	117.0	1.6	93.0	2.7	98.2	1.0	111.2	3.5	65.8	5.5	113.8	9.9	119.4	6.1
	2xLLOV	102.8	1.1	79.8	1.7	83.9	4.3	105.9	2.3	54.5	1.2	113.8	1.8	111.0	2.0
	MOV	99.1	1.9	83.0	1.8	86.7	1.8	101.2	3.8	44.2	2.3	111.8	1.2	114.3	4.7
	ULOV	100.3	0.7	93.1	2.5	93.8	4.7	106.4	4.8	40.6	4.9	113.4	0.8	119.5	3.5
Thalido-mide	LLOV	83.3	4.3	n.a.	n.a.	n.a.	n.a.	69.3	3.5	n.a.	n.a.	98.2	4.1	98.0	2.6
	2xLLOV	78.3	1.1	n.a.	n.a.	n.a.	n.a.	64.9	3.7	n.a.	n.a.	102.3	2.7	101.6	2.1
	MOV	74.7	2.8	n.a.	n.a.	n.a.	n.a.	60.7	0.5	n.a.	n.a.	105.2	1.5	96.2	4.0
	ULOV	72.8	4.1	1.7	0.0	1.7	0.0	55.2	1.7	n.a.	n.a.	96.9	7.7	97.8	3.2
Tivozanib	LLOV	90.0	1.9	99.0	4.4	100.8	2.3	102.5	1.7	38.6	4.2	94.0	2.6	95.0	4.6
	2xLLOV	96.8	2.3	104.0	0.1	103.9	1.6	103.2	2.1	49.4	2.2	101.5	3.3	102.7	2.1
	MOV	99.9	2.3	103.5	0.6	105.8	1.4	100.8	1.7	49.6	0.6	104.6	1.6	102.4	2.9

	ULOV	98.1	1.1	104.7	1.3	103.8	1.4	98.0	0.9	42.5	5.5	101.6	1.2	103.1	1.6
Trametinib	LLOV	93.8	11.0	78.3	3.1	53.1	15.1	90.4	6.1	0.4	2.9	101.7	12.0	100.1	12.6
	2xLLOV	97.6	8.1	88.2	3.0	94.3	3.1	101.9	11.2	40.7	4.1	105.8	8.6	101.8	7.0
	MOV	102.5	6.7	101.6	1.4	99.7	2.3	115.0	3.7	52.0	0.0	103.1	1.1	103.5	5.5
	ULOV	97.0	2.1	96.8	5.0	92.2	4.6	108.2	2.9	47.5	7.5	97.7	5.0	97.0	4.8
Trifluridine	LLOV	80.9	5.3	45.6	2.7	36.1	3.5	83.6	1.1	-89.2	8.9	97.0	5.6	86.9	3.7
	2xLLOV	101.6	2.6	72.7	2.1	76.2	2.9	103.5	2.8	-6.6	12.0	117.8	4.6	107.4	3.0
	MOV	94.4	3.1	78.0	1.6	75.3	1.6	96.3	2.6	28.1	1.9	113.1	0.7	102.7	0.9
	ULOV	91.6	2.1	66.9	1.6	67.3	0.5	85.5	1.8	23.2	3.4	104.4	0.4	98.4	0.5
Vandetanib	LLOV	83.4	1.7	81.4	1.4	80.3	0.2	91.7	1.9	24.9	6.5	85.9	1.1	83.5	1.1
	2xLLOV	97.2	0.8	97.2	1.9	94.8	0.6	99.4	1.6	44.7	2.7	103.5	1.5	102.7	0.9
	MOV	101.5	3.0	95.8	0.4	95.1	1.0	101.6	3.3	49.3	1.7	108.2	1.7	101.8	0.3
	ULOV	94.0	1.9	83.1	2.2	83.3	1.0	88.8	2.2	41.3	3.8	95.3	1.0	94.4	1.4
Vemuraf-enib	LLOV	101.9	3.4	93.0	2.5	82.9	4.9	90.0	6.2	34.3	3.0	112.7	5.1	109.4	2.9
	2xLLOV	98.2	3.8	92.7	2.1	85.9	3.7	93.0	4.9	41.6	3.7	103.5	0.3	102.3	2.7
	MOV	97.5	4.6	89.3	3.0	92.1	2.1	100.4	5.6	39.7	0.6	99.5	1.7	101.8	3.9
	ULOV	99.2	2.2	91.3	1.1	88.2	0.7	93.8	5.4	34.6	4.9	104.1	2.2	99.4	3.4
Venetoclax	LLOV	105.4	4.6	112.5	6.3	102.7	4.3	88.8	3.3	n.a.	n.a.	106.3	1.8	103.3	2.7
	2xLLOV	102.4	8.2	98.6	1.9	95.7	3.3	102.9	6.7	34.5	3.2	106.9	2.9	104.7	1.3
	MOV	97.8	4.7	96.8	3.4	98.0	3.8	100.5	5.2	26.4	1.1	100.0	1.3	102.3	4.1
	ULOV	101.9	3.8	94.1	0.9	92.7	0.3	99.7	4.1	22.4	3.4	103.7	1.2	102.7	4.8
Vinorelbine	LLOV	120.0	6.7	98.2	11.0	96.4	4.0	96.3	9.0	67.0	0.0	65.0	8.0	67.9	2.8
	2xLLOV	105.8	5.6	78.0	3.9	79.3	6.2	88.7	11.8	42.2	1.3	89.9	8.3	87.3	9.7
	MOV	95.7	1.2	66.8	1.6	70.0	1.1	93.6	3.3	37.2	1.1	97.7	0.7	99.6	4.3
	ULOV	96.7	1.3	62.0	0.8	57.4	0.3	91.1	4.5	29.6	3.1	102.4	4.2	99.5	0.9
Vismodegib	LLOV	77.6	2.2	96.1	2.0	99.6	0.7	87.2	3.2	34.2	8.3	84.9	1.7	85.6	2.8
	2xLLOV	103.4	0.9	114.6	0.8	117.6	0.6	105.0	1.2	63.8	3.9	105.1	3.2	109.2	1.0
	MOV	103.2	1.1	111.1	1.1	114.6	0.2	105.3	1.0	65.7	0.7	108.4	0.5	107.5	1.1
	ULOV	94.1	0.6	103.5	0.8	104.5	1.2	94.5	0.5	54.4	5.1	96.6	1.0	99.7	0.6

n.d.: not detectable, ULOV: upper limit of validation, MOV: middle of validation limit, LLOV: lower limit of validation.

Table S7. Long-term stability test results.

Oral an-ticancer drug	Validation standard	Long-term stability, 6 weeks, 4°C, without day light		Long-term stability, 1 month, -80°C		Long-term stability, 1 month, -20°C		Long-term stability, 2 month, -80°C		Long-term stability, 2 month, -20°C		Long-term stability, 3 month, -80°C		Long-term stability, 3 month, -20°C	
		Accuracy [%]	CV [%]	Accuracy [%]	CV [%]	Accu-racy [%]	CV [%]	Accu-racy [%]	CV [%]	Accu-racy [%]	CV [%]	Accu-racy [%]	CV [%]	Accu-racy [%]	CV [%]
Abema-ciclib	LLOV	86.3	1.8	88.4	6.7	103.5	0.5	112.7	3.2	107.2	5.8	101.8	4.8	95.7	7.6
	2xLLOV	92.0	4.8	90.1	2.4	95.0	2.5	111.4	0.5	109.1	6.0	103.4	3.0	98.8	2.1
	MOV	106.3	4.9	96.2	3.0	103.0	4.2	105.5	0.5	107.3	2.6	101.8	1.0	104.4	2.7
	ULOV	99.1	0.9	97.8	4.7	102.4	3.0	105.1	1.8	106.0	0.9	99.9	2.5	103.5	2.8
Abi-raterone	LLOV	110.7	4.3	93.2	1.7	96.5	1.2	105.8	10.1	97.0	3.8	106.0	2.6	101.7	5.7
	2xLLOV	101.6	4.4	93.5	0.6	96.2	3.4	100.1	4.0	101.0	2.3	97.9	1.3	97.2	1.2
	MOV	106.4	3.1	93.5	1.5	95.8	1.6	102.9	3.4	101.2	5.1	100.6	1.5	101.8	2.5
	ULOV	102.8	1.1	93.2	2.1	98.3	0.7	103.2	2.1	102.5	2.2	100.6	1.7	99.7	1.3
Afatinib	LLOV	2.9	2.1	75.4	6.8	70.0	9.0	85.8	9.7	68.4	5.3	90.0	7.0	68.4	2.9
	2xLLOV	26.7	10.6	90.7	1.3	79.7	1.2	92.5	4.0	72.1	3.6	94.1	2.6	76.9	6.2
	MOV	44.2	1.5	93.1	2.1	87.9	1.8	96.0	4.5	84.4	2.6	96.4	0.7	78.2	2.0
	ULOV	37.3	1.9	91.2	0.9	86.5	0.2	96.0	0.6	88.3	4.3	92.1	1.9	82.8	3.2
Alectinib	LLOV	75.7	1.2	83.5	1.3	84.7	3.4	81.8	1.9	79.9	2.1	81.6	1.5	79.0	1.7
	2xLLOV	81.2	0.9	83.3	3.3	87.1	1.3	86.9	1.1	86.4	0.8	86.1	0.4	82.7	2.7
	MOV	92.7	2.9	83.9	0.2	85.8	0.9	89.3	1.6	89.5	1.3	86.7	1.2	88.4	1.6
	ULOV	86.5	0.7	81.7	1.9	85.2	1.1	88.1	1.4	88.3	1.3	84.8	1.6	84.7	1.3

Ana-grelide	LLOV	33.5	4.4	121.5	10.5	103.9	4.2	141.5	10.2	128.7	5.7	118.8	9.6	101.8	15.8
	2xLLOV	74.6	4.6	105.6	7.6	99.8	6.8	118.5	6.0	121.4	3.3	114.9	4.2	110.2	7.0
	MOV	103.7	6.7	97.0	2.9	95.0	4.7	98.7	4.0	101.3	3.4	95.1	5.6	95.7	1.2
	ULOV	97.1	2.6	90.8	1.6	95.4	3.4	99.2	1.9	94.5	1.4	94.7	2.0	94.9	2.5
Apalu-tamide	LLOV	78.5	3.8	100.0	1.2	96.1	4.5	102.3	3.2	93.4	0.9	98.4	2.0	90.4	2.7
	2xLLOV	73.5	1.3	93.2	2.2	94.7	0.8	100.0	2.8	91.6	3.5	94.9	1.0	85.1	1.4
	MOV	54.9	2.3	91.1	3.1	89.4	2.9	97.6	1.3	90.2	9.3	89.7	3.8	86.8	4.3
	ULOV	48.1	1.9	90.2	2.9	90.6	2.3	95.9	1.0	88.9	2.9	90.1	4.3	78.8	3.5
Ax-itinib	LLOV	49.2	1.2	83.07	3.87	87.8	2.9	90.5	20.6	110.1	3.8	102.5	3.0	107.3	9.1
	2xLLOV	66.5	10.7	86.83	7.24	86.8	5.4	94.2	6.7	87.6	5.8	100.1	6.2	92.9	5.4
	MOV	100.5	5.5	87.50	2.56	89.8	2.6	92.2	2.0	87.7	1.5	90.5	3.8	95.9	2.6
	ULOV	91.2	0.3	87.13	2.23	92.0	0.7	91.3	3.5	88.8	4.5	91.9	1.0	86.8	3.3
Binimeti nib	LLOV	104.1	8.0	106.3	11.8	103.4	8.5	124.3	3.1	116.3	2.8	104.6	6.9	105.4	5.0
	2xLLOV	95.3	4.1	97.4	4.6	94.8	2.3	106.4	4.5	105.7	2.7	104.4	6.6	99.5	3.4
	MOV	109.9	4.5	94.4	1.1	92.3	1.2	96.6	1.2	95.9	2.2	98.7	4.2	97.9	1.7
	ULOV	99.7	0.8	97.2	0.7	99.6	1.5	101.1	4.4	99.2	5.0	102.6	0.5	98.9	1.9
Bosu-tinib	LLOV	80.7	9.2	99.7	7.0	104.4	6.4	98.4	4.1	73.8	6.8	90.4	5.8	83.9	0.9
	2xLLOV	84.2	7.1	100.4	4.9	105.5	4.5	106.2	4.1	75.3	15.6	98.2	4.5	68.1	8.3
	MOV	98.0	3.4	102.3	5.0	95.2	7.0	103.3	3.3	98.0	12.9	108.4	5.4	108.6	7.3
	ULOV	90.3	1.2	98.7	6.3	100.5	0.9	105.6	5.2	107.7	2.7	104.6	5.1	106.7	3.2
Brigat-inib	LLOV	89.6	3.4	88.3	6.2	93.6	3.5	96.7	13.5	100.6	4.4	93.0	2.7	92.1	1.1
	2xLLOV	94.2	3.1	92.1	1.4	92.4	5.2	110.2	2.6	105.4	6.4	99.0	4.4	96.3	2.4
	MOV	104.7	2.6	96.9	2.4	100.8	2.8	107.5	1.2	108.5	5.0	106.8	4.8	105.5	2.0
	ULOV	98.6	1.2	95.0	5.1	100.4	2.5	108.0	1.8	105.8	4.1	98.7	1.9	102.6	2.8
Cabozan tinib	LLOV	94.1	1.3	89.4	1.1	91.9	1.3	100.2	1.8	97.7	2.0	91.8	0.7	90.4	1.6
	2xLLOV	96.8	2.5	92.5	2.8	98.4	1.9	100.3	2.7	100.6	1.8	94.6	1.1	92.5	2.6
	MOV	108.5	2.9	97.3	0.6	97.7	2.1	102.9	0.5	101.6	1.2	98.9	0.6	100.0	1.6
	ULOV	101.8	1.6	93.1	2.4	95.7	1.4	100.3	1.5	99.6	0.9	93.1	2.0	96.3	2.9
Ceritinib	LLOV	78.9	1.7	96.1	0.1	101.5	3.2	103.7	1.9	99.7	2.7	100.3	1.5	96.9	1.7
	2xLLOV	77.8	0.5	89.5	1.6	94.3	2.1	96.4	1.1	94.4	2.0	93.4	1.6	91.8	5.0
	MOV	104.9	3.1	93.7	1.3	93.3	1.4	95.5	1.4	94.6	0.8	95.7	1.7	94.1	1.2
	ULOV	97.9	1.9	94.0	1.4	97.3	1.7	100.5	3.6	99.1	2.3	100.0	0.8	101.8	1.0
Cobi-metinib	LLOV	94.5	1.6	99.0	3.3	101.1	2.8	97.9	4.0	99.0	2.8	100.3	2.2	101.3	4.7
	2xLLOV	94.8	1.8	93.9	3.4	100.8	1.8	103.4	5.8	98.0	1.0	97.6	1.9	99.0	0.7
	MOV	108.3	4.2	96.8	0.7	98.9	1.7	103.3	1.1	100.8	1.0	98.4	1.7	99.2	1.6
	ULOV	102.4	2.7	97.3	2.7	100.6	0.9	103.6	2.2	104.5	2.7	98.3	2.9	100.6	4.2
Crizo-tinib	LLOV	58.2	2.0	75.4	3.2	77.1	1.4	78.9	2.0	74.2	1.4	73.9	3.4	72.6	2.8
	2xLLOV	68.4	3.4	85.4	2.4	87.5	2.0	93.8	0.5	90.8	5.0	90.2	3.7	87.5	1.7
	MOV	93.4	2.9	89.3	1.4	90.2	2.8	96.2	2.4	96.3	1.3	95.1	1.5	94.9	3.5
	ULOV	83.8	1.4	80.1	1.2	84.4	2.7	89.2	0.8	88.8	0.8	89.7	2.1	88.4	2.0
Dabraf-enib	LLOV	102.4	1.2	96.1	2.9	101.0	4.5	97.9	3.5	99.0	3.7	96.6	2.7	97.1	3.1
	2xLLOV	107.2	3.6	93.1	1.2	101.2	3.3	101.3	2.8	98.6	1.7	101.7	3.2	96.6	1.2
	MOV	107.2	5.9	92.3	1.7	96.1	1.7	98.9	2.6	97.8	2.9	98.0	0.7	100.3	0.5
	ULOV	100.6	1.3	92.2	1.7	97.0	0.4	98.0	0.2	97.4	1.1	98.6	1.2	96.3	1.0
Dacomiti nib	LLOV	61.0	1.8	87.2	3.8	86.5	1.7	105.1	3.3	96.8	4.3	96.1	2.8	83.4	3.4
	2xLLOV	62.3	0.4	91.2	1.4	94.4	3.3	107.1	1.2	96.1	5.2	94.7	4.8	89.4	5.8
	MOV	79.1	1.7	95.2	1.6	91.7	1.2	99.2	2.5	95.1	3.7	99.4	2.4	90.9	2.9
	ULOV	77.8	4.4	93.2	3.3	93.0	1.4	99.6	2.2	96.3	2.2	97.6	3.0	90.3	5.7
Da-satinib	LLOV	79.4	3.5	89.0	8.8	108.6	9.4	84.9	10.2	82.2	6.2	85.5	4.6	81.3	8.7
	2xLLOV	72.6	3.9	92.5	2.5	99.2	3.4	96.3	4.9	98.2	11.7	99.1	6.1	102.0	10.3
	MOV	102.3	6.4	91.3	2.2	97.7	0.3	92.3	3.5	92.8	5.3	101.1	4.2	102.9	3.9
	ULOV	97.9	2.5	93.4	2.4	97.7	2.2	100.4	0.9	95.6	3.2	105.8	3.8	104.5	2.2
Encoraf-enib	LLOV	81.0	9.4	88.1	5.17	84.2	4.0	93.1	5.4	103.4	10.7	94.9	2.8	96.1	5.9
	2xLLOV	95.2	9.4	93.5	0.73	100.7	3.2	104.1	6.6	111.7	5.5	95.2	2.8	97.4	7.1
	MOV	109.2	4.1	97.1	3.47	99.0	0.7	104.9	3.0	102.3	2.1	100.8	1.2	101.8	3.3
	ULOV	101.7	0.6	95.7	3.36	99.1	2.9	101.8	3.2	104.0	1.7	99.0	3.9	100.7	1.8

Enzalu-tamide	LLOV	97.2	0.8	82.3	11.2	92.6	2.7	94.8	0.7	90.2	1.2	88.4	1.7	88.7	1.5
	2xLLOV	96.8	2.9	87.8	3.7	92.7	1.5	92.9	2.6	91.5	2.3	89.0	3.0	87.1	0.5
	MOV	105.1	2.5	90.6	1.1	90.3	0.4	95.7	1.3	92.8	2.6	90.6	1.6	91.6	0.5
	ULOV	95.0	1.5	86.1	1.3	85.8	1.3	90.3	3.3	90.8	3.3	87.3	2.7	87.7	4.7
Erlotinib	LLOV	107.0	0.8	108.0	2.0	113.5	2.0	114.8	0.9	113.2	0.7	114.1	3.4	111.5	6.2
	2xLLOV	95.9	3.1	98.1	1.9	102.3	2.2	105.9	0.5	106.4	0.9	105.8	0.6	106.0	3.7
	MOV	111.0	4.1	97.0	1.6	97.4	1.5	101.3	3.1	102.2	3.4	102.8	2.0	102.2	2.3
	ULOV	103.7	0.9	97.8	1.2	99.6	0.9	104.4	2.5	105.2	4.4	104.4	1.1	101.8	4.5
Gefitinib	LLOV	78.5	0.9	89.0	2.1	95.8	1.8	102.7	1.2	101.3	1.6	99.5	1.0	100.2	3.6
	2xLLOV	89.3	3.5	94.2	1.0	99.2	2.5	108.5	1.7	105.6	2.9	101.3	2.0	103.9	2.7
	MOV	105.7	2.9	99.3	1.2	100.7	2.9	104.1	3.3	105.1	1.3	99.9	2.3	102.7	1.6
	ULOV	97.6	2.1	93.5	0.4	98.5	1.9	101.3	1.1	99.2	0.5	99.2	2.7	99.2	3.4
Ibrutinib	LLOV	47.2	2.6	95.4	3.3	92.5	1.8	108.9	3.0	99.6	1.4	97.4	1.3	88.6	0.8
	2xLLOV	41.2	1.5	92.3	3.5	97.3	2.9	100.9	1.3	97.7	2.3	96.2	2.0	87.9	3.8
	MOV	36.1	1.0	93.6	1.6	94.1	2.5	101.3	1.2	97.4	2.9	95.3	1.5	90.4	4.0
	ULOV	44.1	0.3	92.1	2.8	93.3	2.8	101.9	3.1	99.3	1.6	95.0	1.1	86.9	3.0
Idelalisi b	LLOV	113.3	0.9	107.6	2.6	113.2	1.3	117.1	2.2	114.5	2.3	111.2	1.5	106.7	4.8
	2xLLOV	99.6	2.6	96.4	1.8	99.5	1.9	104.0	1.7	103.3	1.9	106.2	0.7	100.7	3.3
	MOV	107.2	4.4	94.7	0.6	95.7	1.8	97.4	1.8	98.7	2.2	102.2	1.5	102.8	1.8
	ULOV	102.9	1.7	96.0	1.4	100.0	2.7	103.3	1.0	101.9	3.4	104.3	1.1	103.1	2.5
Imatinib	LLOV	86.4	6.4	77.3	9.9	88.0	3.1	103.3	0.9	95.6	3.1	18.2	5.9	19.7	4.1
	2xLLOV	103.4	3.6	96.3	1.9	97.8	2.3	112.9	4.6	104.1	11.3	72.7	12.0	64.1	6.2
	MOV	95.4	3.6	84.4	2.2	87.1	0.6	96.4	3.6	99.4	4.6	90.1	2.6	90.0	2.3
	ULOV	89.8	3.2	79.8	3.4	87.9	0.2	89.1	1.0	91.0	2.1	89.4	3.6	89.6	1.7
Lapa-tinib	LLOV	28.2	0.8	91.6	2.0	85.5	2.3	98.5	2.0	80.7	2.4	95.3	0.5	55.4	9.8
	2xLLOV	34.8	1.1	92.2	2.3	91.4	4.3	102.3	1.1	84.4	2.9	96.3	1.1	72.0	16.7
	MOV	101.1	3.6	97.6	1.4	95.3	2.2	103.0	1.0	93.7	8.4	101.0	1.2	78.1	13.3
	ULOV	99.9	1.7	94.5	1.6	96.6	0.9	100.9	2.5	101.5	1.8	96.7	1.5	95.4	3.9
Larotrec-tinib	LLOV	115.2	9.7	87.5	0.6	85.6	0.6	102.1	3.0	103.5	7.4	94.2	2.4	96.4	2.6
	2xLLOV	106.7	7.6	92.9	5.5	97.9	2.9	104.7	4.4	103.6	1.5	98.8	1.2	100.8	1.0
	MOV	105.2	0.8	97.2	3.2	96.7	1.2	103.2	0.9	99.1	1.8	91.1	3.7	96.2	1.7
	ULOV	98.0	3.3	95.5	1.4	95.0	2.0	104.6	3.1	102.5	2.8	95.6	3.0	97.8	2.4
Lenalid-omide	LLOV	n.a.	n.a.	96.6	6.6	104.5	5.4	56.2	2.5	52.8	4.2	82.4	8.0	69.1	7.9
	2xLLOV	15.8	1.3	103.2	2.8	112.2	1.8	104.0	6.0	97.9	3.2	104.1	3.0	99.5	2.8
	MOV	19.7	0.9	93.9	1.9	93.9	1.9	104.0	3.6	101.8	2.5	96.0	1.1	92.9	1.9
	ULOV	16.2	3.1	88.5	1.8	92.6	1.4	98.8	2.4	95.8	1.4	94.4	0.9	89.8	2.2
Len-vatinib	LLOV	127.4	5.0	107.0	4.7	109.4	4.5	108.7	4.5	102.7	1.2	110.3	3.6	105.7	2.6
	2xLLOV	105.2	6.7	94.9	1.9	99.7	2.3	100.9	1.1	97.0	4.2	104.8	2.0	103.0	5.5
	MOV	107.3	4.0	94.5	1.4	95.8	1.0	97.2	4.5	98.2	2.5	100.5	2.1	99.7	1.1
	ULOV	101.9	2.6	94.7	1.9	96.4	0.8	101.9	2.8	101.7	4.8	100.8	1.2	98.3	3.2
Lorla-tinib	LLOV	97.1	2.0	82.6	3.1	87.9	0.3	87.6	0.5	90.0	1.4	93.2	1.1	95.7	1.1
	2xLLOV	96.7	2.3	84.9	1.1	89.2	0.8	91.9	1.3	91.9	3.5	90.8	2.1	91.7	0.6
	MOV	93.7	1.7	87.8	0.6	88.5	1.0	91.2	2.4	93.9	3.1	89.2	0.9	90.9	2.2
	ULOV	91.4	2.1	87.4	0.9	90.6	1.5	91.7	0.5	91.4	1.4	89.2	0.5	91.0	2.4
Midosta urin	LLOV	102.3	3.5	106.4	10.8	109.4	3.1	101.2	6.0	106.5	4.1	112.8	4.6	102.8	8.7
	2xLLOV	94.2	1.6	106.8	3.5	105.5	3.3	108.5	4.9	106.0	6.8	106.8	1.5	97.1	5.2
	MOV	103.5	1.7	108.1	6.3	105.8	2.4	116.3	7.0	113.7	7.3	102.9	4.1	102.0	5.4
	ULOV	97.3	1.8	103.4	1.1	107.3	2.6	111.4	6.8	118.6	8.1	107.0	3.6	93.8	6.5
Nerat-inib	LLOV	n.a.	n.a.	71.8	2.0	66.9	5.7	88.3	2.3	77.1	8.6	81.4	4.6	50.3	4.9
	2xLLOV	7.4	0.4	84.9	2.7	79.9	9.1	94.6	7.8	67.5	6.1	90.2	11.9	70.3	6.8
	MOV	10.0	1.1	84.6	1.5	82.9	1.1	92.9	3.5	81.2	5.2	92.5	1.3	79.5	6.5
	ULOV	8.5	0.3	78.2	1.2	79.7	1.0	88.3	3.9	82.8	2.3	91.8	1.3	75.5	4.3
Nilotinib	LLOV	102.0	2.6	89.8	1.9	91.9	0.8	97.4	1.5	93.3	1.2	93.1	1.3	90.5	3.6
	2xLLOV	103.4	2.7	89.2	2.2	95.1	1.0	99.7	1.9	96.9	0.9	92.2	1.3	91.6	1.8
	MOV	101.7	3.8	91.9	2.0	95.6	1.7	98.0	2.1	99.1	2.0	93.1	2.0	96.9	2.5
	ULOV	98.0	1.2	90.9	1.7	94.2	1.1	97.0	2.4	97.5	1.6	91.6	2.3	93.1	3.0

Nintedanib	LLOV	86.6	10.7	102.1	4.6	102.0	1.8	94.3	4.4	96.9	9.1	88.3	3.5	88.2	1.7
	2xLLOV	94.4	5.3	98.9	4.4	97.2	5.9	99.7	4.6	103.8	4.0	91.1	0.8	90.3	4.1
	MOV	94.4	0.4	91.4	0.4	95.5	2.2	99.7	1.0	98.9	2.1	94.7	0.6	97.2	3.3
	ULOV	85.9	1.5	87.4	1.6	94.5	0.8	94.8	2.4	97.0	0.6	91.8	1.2	92.9	2.0
Niraparib	LLOV	94.3	1.5	93.4	2.3	97.8	1.6	102.4	0.9	101.8	0.7	95.4	0.7	96.1	1.8
	2xLLOV	96.1	1.2	94.5	1.1	101.4	0.9	106.0	1.3	104.1	2.1	95.7	2.6	99.8	1.8
	MOV	104.5	3.4	99.6	1.6	99.8	0.3	103.9	2.1	105.3	2.6	100.4	1.3	102.2	1.9
	ULOV	96.4	2.4	98.5	1.7	100.8	1.9	104.1	0.5	103.3	1.4	101.8	1.5	100.4	4.6
Olaparib	LLOV	105.6	0.4	110.5	0.7	112.8	0.5	115.8	1.0	115.9	0.5	115.4	0.9	111.4	3.3
	2xLLOV	96.6	1.4	98.2	0.7	100.3	0.5	103.3	0.5	103.8	2.0	103.3	0.3	99.8	1.6
	MOV	107.5	5.2	92.9	1.1	95.3	0.8	96.8	1.6	97.5	1.6	96.7	1.3	97.6	1.3
	ULOV	105.6	0.7	98.1	2.2	99.1	2.0	101.1	1.9	102.1	2.6	100.7	1.0	98.9	1.2
Osimertinib	LLOV	5.4	1.3	86.9	0.7	83.0	6.8	96.9	5.0	84.8	3.7	91.8	4.7	66.7	3.4
	2xLLOV	10.5	1.6	93.2	1.5	95.2	2.7	103.9	3.4	89.2	0.9	100.0	2.1	82.8	6.7
	MOV	53.4	1.7	96.6	2.0	97.0	3.4	101.6	1.2	95.2	1.2	101.8	2.6	90.9	5.1
	ULOV	41.5	2.4	93.2	2.8	92.3	3.8	101.4	1.4	91.4	0.5	93.8	1.7	88.6	5.2
Palbociclib	LLOV	75.6	2.0	85.3	4.5	88.0	0.8	105.3	3.4	99.0	4.5	94.4	0.8	89.2	4.7
	2xLLOV	75.5	2.6	90.8	1.4	92.5	1.2	103.1	2.6	99.9	2.4	99.4	5.8	96.8	4.0
	MOV	89.0	2.8	93.0	1.9	95.4	0.4	98.2	2.4	96.5	1.8	102.2	2.3	94.2	3.4
	ULOV	88.7	2.7	93.0	1.0	98.4	2.3	97.1	0.3	96.9	1.4	104.8	0.6	104.4	3.6
Panobinostat	LLOV	n.a.	n.a.	33.7	13.9	38.2	5.9	29.9	30.2	21.2	40.8	34.0	10.7	8.2	13.5
	2xLLOV	11.4	3.1	59.0	9.5	52.2	2.8	107.6	5.4	66.3	18.5	67.6	4.8	57.5	8.3
	MOV	67.6	0.7	84.1	0.8	82.0	2.1	87.5	9.1	75.7	8.3	82.1	1.6	66.1	9.5
	ULOV	79.4	0.9	89.9	1.7	89.6	4.5	99.4	3.5	98.1	0.9	100.2	1.8	87.5	2.2
Pazopanib	LLOV	99.0	0.6	97.4	1.1	102.3	3.3	108.3	1.0	106.7	3.6	103.6	2.3	103.9	3.6
	2xLLOV	96.5	2.5	95.8	3.9	99.1	0.9	103.8	1.6	100.4	2.2	104.8	1.1	106.9	3.0
	MOV	109.7	3.9	95.8	0.6	96.3	1.0	98.1	2.6	98.8	2.1	103.3	0.4	103.3	0.7
	ULOV	98.9	1.4	93.0	1.7	97.3	0.5	98.3	1.8	98.8	4.8	100.0	0.7	98.1	2.4
Pomalidomide	LLOV	30.1	0.8	85.8	6.5	91.3	1.4	99.9	12.7	95.9	11.7	97.4	10.1	97.6	10.6
	2xLLOV	19.0	3.6	90.2	3.5	90.4	4.6	96.6	3.8	107.3	9.1	97.4	3.2	95.5	1.2
	MOV	15.7	1.2	88.3	0.6	90.6	1.4	99.4	1.8	97.2	3.9	94.9	1.7	90.0	0.4
	ULOV	11.3	2.3	89.1	0.8	94.6	0.9	99.0	2.5	94.1	0.6	95.1	1.8	87.6	1.5
Ponatinib	LLOV	83.5	0.8	92.6	1.7	96.0	4.1	99.1	3.3	87.7	0.3	96.8	2.5	91.3	3.0
	2xLLOV	91.7	2.3	94.7	2.7	99.2	2.9	97.8	3.5	95.8	3.2	98.9	2.8	93.4	5.8
	MOV	97.9	4.5	93.7	1.2	92.3	2.1	101.1	1.2	99.2	1.0	95.3	3.0	95.0	1.7
	ULOV	89.4	2.0	90.8	2.4	95.0	0.5	100.1	1.8	97.0	1.1	93.5	0.2	93.8	3.0
Regorafenib	LLOV	79.3	2.1	102.9	3.6	105.4	2.6	104.7	7.1	106.3	1.4	105.1	3.0	94.0	5.5
	2xLLOV	80.6	2.8	104.0	5.5	101.2	1.2	104.3	3.5	105.6	4.9	103.7	3.7	90.7	5.9
	MOV	101.0	2.9	106.3	3.4	101.6	1.0	112.2	6.7	105.8	5.9	100.4	6.4	97.1	6.1
	ULOV	95.5	0.9	103.8	1.3	104.7	3.1	107.0	5.4	108.5	7.8	103.1	4.2	92.9	8.7
Ribociclib	LLOV	76.6	2.8	83.5	2.4	88.0	1.9	94.7	2.2	92.7	1.6	90.7	1.8	89.1	2.5
	2xLLOV	96.4	1.2	98.5	1.2	101.7	1.9	112.4	2.4	109.0	2.7	106.4	3.0	105.3	0.4
	MOV	107.4	1.0	101.3	1.1	101.6	2.1	108.1	2.2	108.6	1.7	104.3	1.7	102.5	1.3
	ULOV	96.3	1.4	90.7	1.0	91.9	3.0	98.8	0.5	98.7	1.5	92.7	2.0	93.9	2.8
Rucaparib	LLOV	104.4	1.3	102.1	1.2	104.7	0.2	112.2	3.6	115.1	4.0	108.0	1.8	109.6	0.7
	2xLLOV	93.1	1.5	89.7	0.2	95.4	0.5	99.7	3.7	100.7	3.8	94.7	1.8	95.9	0.9
	MOV	103.5	1.2	95.5	1.0	98.5	2.8	102.1	2.8	104.6	2.5	97.2	0.7	101.6	1.0
	ULOV	100.2	1.8	94.8	0.2	99.8	1.5	102.7	1.1	104.3	0.2	98.3	2.4	100.6	3.9
Ruxolitinib	LLOV	97.9	2.4	94.6	1.4	95.6	2.5	99.8	7.1	98.6	2.6	96.5	3.9	101.8	5.8
	2xLLOV	93.8	2.2	93.0	2.8	101.5	3.6	100.0	1.4	97.7	0.6	99.4	3.8	97.5	0.5
	MOV	97.6	2.6	94.2	2.3	95.8	1.4	96.8	2.0	97.7	0.5	94.6	0.6	97.0	1.3
	ULOV	96.6	2.1	91.6	1.4	95.3	2.9	95.8	0.9	95.3	0.3	94.5	1.0	95.4	2.8
Sonidegib	LLOV	89.3	3.8	108.6	3.1	109.9	0.5	104.4	5.9	105.8	5.6	109.9	2.7	103.1	4.0
	2xLLOV	91.2	3.8	108.9	3.9	106.2	1.8	107.0	2.0	111.0	3.1	104.6	5.3	96.1	3.3
	MOV	106.1	2.3	108.0	4.5	104.9	4.6	119.3	6.6	114.5	5.0	99.2	3.3	95.1	5.3
	ULOV	100.7	2.4	105.4	0.5	106.5	3.9	122.3	9.9	122.3	9.9	100.0	4.7	89.3	6.8

Soraf-enib	LLOV	71.1	2.5	102.7	3.3	106.1	1.7	106.5	4.2	109.0	3.9	111.4	4.5	100.5	2.0
	2xLLOV	74.5	2.6	110.1	2.6	103.7	1.5	106.2	3.7	109.9	3.3	108.7	4.2	94.5	3.7
	MOV	106.5	3.6	108.3	4.5	106.1	5.3	118.4	4.6	112.2	2.6	100.4	4.3	94.0	6.2
	ULOV	99.7	3.7	103.5	0.4	104.5	3.3	114.3	6.8	116.0	7.6	102.3	5.4	90.5	5.4
Sunitinib	LLOV	96.2	1.9	122.7	2.1	132.8	3.0	112.8	5.3	114.3	3.5	135.5	3.4	130.7	6.5
	2xLLOV	85.9	4.2	108.5	3.0	121.2	1.4	108.9	3.6	108.9	5.5	131.1	4.5	123.9	3.2
	MOV	109.9	5.2	107.0	1.9	118.5	5.8	102.8	3.4	106.3	4.9	120.9	0.6	123.6	1.6
	ULOV	105.4	1.1	109.0	1.8	119.5	3.7	108.8	5.5	109.7	7.0	118.4	4.6	122.5	5.4
Thalido-mide	LLOV	n.a.	n.a.	76.7	2.8	71.4	3.7	72.7	1.3	57.5	7.6	76.7	3.0	43.9	4.7
	2xLLOV	n.a.	n.a.	74.9	3.1	75.6	7.6	79.7	5.7	58.1	7.2	79.8	7.0	50.7	10.4
	MOV	n.a.	n.a.	73.1	4.3	66.7	8.6	77.9	2.4	57.0	16.2	75.0	1.3	50.8	12.0
	ULOV	n.a.	n.a.	66.3	0.3	68.6	6.9	79.5	2.8	42.5	4.3	78.0	4.7	33.5	6.9
Tivoza-nib	LLOV	86.9	4.0	96.0	2.0	99.8	1.6	100.7	1.3	95.8	1.6	97.1	1.5	92.5	0.8
	2xLLOV	93.9	4.1	95.7	2.6	101.4	3.0	101.4	4.2	98.9	2.3	95.2	0.9	96.9	4.5
	MOV	107.6	1.9	95.0	0.8	97.4	0.3	101.3	0.8	102.0	2.4	98.0	1.4	100.0	0.7
	ULOV	100.6	0.9	92.8	2.7	97.0	2.4	100.3	2.3	100.9	1.9	96.2	3.5	96.3	1.6
Tramet-inib	LLOV	101.5	13.4	101.9	11.2	111.6	4.6	103.4	5.9	111.6	5.5	104.8	5.6	96.6	7.8
	2xLLOV	97.4	0.6	110.9	2.9	105.1	4.2	104.2	6.2	123.9	10.2	103.8	1.5	105.5	1.0
	MOV	103.9	3.8	107.1	3.6	105.2	6.8	117.6	9.9	116.1	6.0	102.5	2.9	102.9	6.1
	ULOV	94.7	4.6	99.6	0.7	103.2	4.1	115.3	5.9	119.8	8.1	106.9	2.3	94.5	6.6
Tri-fluridine	LLOV	49.1	6.8	95.2	4.5	97.0	3.3	70.3	6.5	79.5	3.3	85.8	2.5	89.9	4.1
	2xLLOV	68.8	1.8	105.0	1.7	112.0	2.5	104.3	1.9	107.3	5.9	99.5	1.7	103.6	1.3
	MOV	59.0	0.9	94.6	0.6	99.4	1.5	104.7	2.7	109.3	3.2	90.8	1.3	92.2	0.8
	ULOV	27.1	1.1	88.8	0.7	93.7	2.4	95.5	0.5	98.4	0.4	79.9	0.9	82.1	2.0
Vandeta nib	LLOV	85.4	1.4	88.9	1.9	95.3	1.4	97.6	2.9	97.5	1.8	83.6	2.5	87.6	1.9
	2xLLOV	92.2	2.4	97.7	0.8	99.7	2.1	106.4	2.3	105.9	2.7	101.7	4.9	101.7	2.6
	MOV	100.6	3.1	95.9	1.1	98.6	2.5	105.2	2.8	105.7	2.8	104.4	1.6	105.3	3.4
	ULOV	93.5	1.7	88.0	1.1	93.1	2.5	98.6	1.4	98.6	0.2	98.8	1.4	97.4	2.9
Vemu-rafenib	LLOV	85.9	1.0	101.6	6.0	105.8	2.1	98.7	4.0	104.5	6.1	114.9	3.1	106.2	2.9
	2xLLOV	86.3	3.9	105.7	4.0	102.1	2.7	108.1	4.3	110.6	7.7	105.9	3.6	97.3	6.2
	MOV	97.8	1.5	106.7	6.8	104.9	3.7	119.1	6.4	116.1	5.2	96.8	5.4	92.9	4.3
	ULOV	94.4	1.8	101.7	1.3	104.7	4.2	112.7	6.5	119.0	7.5	100.8	3.4	89.5	6.0
Veneto-clax	LLOV	92.7	7.8	109.1	9.2	111.0	4.2	115.7	20.1	105.5	9.3	99.3	9.2	101.9	7.6
	2xLLOV	99.8	5.1	104.0	3.8	102.9	0.7	111.9	4.1	104.9	2.2	101.9	2.7	85.1	4.1
	MOV	99.5	3.5	103.6	5.8	99.9	3.9	110.0	5.6	109.3	9.3	99.6	4.7	100.0	5.5
	ULOV	95.2	1.2	102.7	0.3	108.0	4.5	114.0	7.3	112.4	7.1	102.2	2.6	92.9	6.8
Vi-norelbine	LLOV	85.9	7.4	110.0	8.3	118.3	16.7	109.4	14.1	98.1	7.7	118.7	6.5	101.7	5.7
	2xLLOV	75.3	11.3	110.2	7.4	97.3	4.1	94.1	7.7	108.5	1.1	97.8	7.2	98.4	4.9
	MOV	73.1	4.1	88.9	3.0	90.2	2.3	92.4	2.1	89.2	5.9	93.5	0.4	98.2	9.0
	ULOV	56.9	2.7	92.0	3.2	90.4	6.4	99.6	1.7	87.6	3.1	98.1	3.7	92.2	6.4
Vismo-degib	LLOV	96.6	1.0	88.6	0.5	92.5	1.1	96.9	1.9	94.1	1.4	84.4	0.9	84.3	2.2
	2xLLOV	107.5	2.1	99.5	1.0	105.4	0.6	104.3	3.0	106.5	2.1	99.6	0.9	100.1	1.8
	MOV	109.8	1.6	97.9	0.6	97.8	1.9	103.7	0.7	103.0	2.8	100.9	1.1	101.3	1.1
	ULOV	96.5	1.7	87.8	1.7	90.5	0.6	95.1	1.6	94.5	1.2	93.4	2.2	94.9	1.7

n.d.: not detectable, ULOV: upper limit of validation, MOV: middle of validation limit, LLOV: lower limit of validation.

References

1. Food and Drug Administration. Center for drug evaluation and research. Abemaciclib. Multi-discipline review. Available online: https://www.accessdata.fda.gov/drugsatfda_docs/nda/2017/208716Orig1s000MultidisciplineR.pdf (accessed on 24 September 2021).
2. Mueller-Schoell, A.; Groenland, S.L.; Scherf-Clavel, O.; van Dyk, M.; Huisinga, W.; Michelet, R.; Jaehde, U.; Steeghs, N.; Huitema, A.D.R.; Kloft, C. Therapeutic drug monitoring of oral targeted antineoplastic drugs. *Eur. J. Clin. Pharmacol.* **2021**, *77*, 441-464, https://doi.org/10.1007/s00228-020-03014-8.
3. van Nuland, M.; Groenland, S.L.; Bergman, A.M.; Steeghs, N.; Rosing, H.; Venekamp, N.; Huitema, A.D.R.; Beijnen, J.H. Exposure-response analyses of abiraterone and its metabolites in real-world patients with metastatic castration-resistant prostate cancer. *Prostate Cancer Prostatic Dis.* **2020**, *23*, 244-251, https://doi.org/10.1038/s41391-019-0179-5.
4. Food and Drug Administration. Center for drug evaluation and research. Afatinib. Clinical pharmacology and biopharmaceutics review(s). Available online: https://www.accessdata.fda.gov/drugsatfda_docs/nda/2013/201292Orig1s000ClinPharmR.pdf (accessed on 24 September 2021).
5. Verheijen, R.B.; Yu, H.; Schellens, J.H.M.; Beijnen, J.H.; Steeghs, N.; Huitema, A.D.R. Practical recommendations for therapeutic drug monitoring of kinase inhibitors in oncology. *Clin. Pharmacol. Ther.* **2017**, *102*, 765-776, https://doi.org/10.1002/cpt.787.
6. Food and Drug Administration. Center for drug evaluation and research. Alectinib. Clinical Pharmacology and biopharmaceutics review(s). Available online: https://www.accessdata.fda.gov/drugsatfda_docs/nda/2015/208434Orig1s000ClinPharmR.pdf (accessed on 24 September 2021).
7. Shire Pharma Canada ULC. Product monograph Agrylin®. Available online: https://pdf.hres.ca/dpd_pm/00053558.PDF (accessed on 24 September 2021).
8. Food and Drug Administration. Center for drug evaluation and research. Apalutamide. Multi-discipline review. Available online: https://www.accessdata.fda.gov/drugsatfda_docs/nda/2018/210951Orig1s000MultidisciplineR.pdf (accessed on 24 September 2021).
9. Tsuchiya, N.; Igarashi, R.; Suzuki-Honma, N.; Fujiyama, N.; Narita, S.; Inoue, T.; Saito, M.; Akihama, S.; Tsuruta, H.; Miura, M.; et al. Association of pharmacokinetics of axitinib with treatment outcome and adverse events in advanced renal cell carcinoma patients. *J. Clin. Oncol.* **2015**, *33*, 506-506, https://doi.org/10.1200/jco.2015.33.7_suppl.506.
10. Food and Drug Administration. Center for drug evaluation and research. Binimetinib. Multi-discipline review. Available online: https://www.accessdata.fda.gov/drugsatfda_docs/nda/2018/210498Orig1s000MultidisciplineR.pdf (accessed on 24 September 2021).
11. Hsyu, P.-H.; Mould, D.R.; Upton, R.N.; Amantea, M. Pharmacokinetic-pharmacodynamic relationship of bosutinib in patients with chronic phase chronic myeloid leukemia. *Cancer Chemother. Pharmacol.* **2013**, *71*, 209-218, https://doi.org/10.1007/s00280-012-1998-4.
12. Food and Drug Administration. Center for drug evaluation and research. Brigatinib. Multi-discipline review. Available online: https://www.accessdata.fda.gov/drugsatfda_docs/nda/2017/208772Orig1s000MultidisciplineR.pdf (accessed on 24 September 2021).
13. Food and Drug Administration. Center for drug evaluation and research. Cabozantinib. Clinical pharmacology and biopharmaceutics review(s). Available online: https://www.accessdata.fda.gov/drugsatfda_docs/nda/2016/208692Orig1s000ClinPharmR.pdf (accessed on 24 September 2021).
14. Food and Drug Administration. Center for drug evaluation and research. Ceritinib. Clinical pharmacology and biopharmaceutics review(s). Available online: https://www.accessdata.fda.gov/drugsatfda_docs/nda/2014/205755Orig1s000ClinPharmR.pdf (accessed on 24 September 2021).
15. Food and Drug Administration. Center for drug evaluation and research. Cobimetinib. Clinical pharmacology and biopharmaceutics review(s). Available online: https://www.accessdata.fda.gov/drugsatfda_docs/nda/2015/206192Orig1s000ClinPharmR.pdf (accessed on 24 September 2021).
16. Ou, S.H.; Bartlett, C.H.; Mino-Kenudson, M.; Cui, J.; Iafrate, A.J. Crizotinib for the treatment of ALK-rearranged non-small cell lung cancer: a success story to usher in the second decade of molecular targeted therapy in oncology. *Oncologist* **2012**, *17*, 1351-1375, https://doi.org/10.1634/theoncologist.2012-0311.
17. Ouellet, D.; Gibiansky, E.; Leonowens, C.; O'Hagan, A.; Haney, P.; Switzky, J.; Goodman, V.L. Population pharmacokinetics of dabrafenib, a BRAF inhibitor: effect of dose, time, covariates, and relationship with its metabolites. *J. Clin. Pharmacol.* **2014**, *54*, 696-706, https://doi.org/10.1002/jcph.263.
18. Food and Drug Administration. Center for drug evaluation and research. Dacomitinib. Multi-discipline review. Available online: https://www.accessdata.fda.gov/drugsatfda_docs/nda/2018/211288Orig1s000MultidisciplineR.pdf (accessed on 24 September 2021).
19. Wang, X.; Roy, A.; Hochhaus, A.; Kantarjian, H.M.; Chen, T.T.; Shah, N.P. Differential effects of dosing regimen on the safety and efficacy of dasatinib: retrospective exposure-response analysis of a Phase III study. *Clin. Pharmacol.* **2013**, *5*, 85-97, https://doi.org/10.2147/CPAA.S42796.

20. Food and Drug Administration. Center for drug evaluation and research. Encorafenib. Multi-discipline review. Available online: https://www.accessdata.fda.gov/drugsatfda_docs/nda/2018/210496Orig1s000MultidisciplineR.pdf (accessed on 24 September 2021).
21. Food and Drug Administration. Center for drug evaluation and research. Enzalutamide. Clinical pharmacology and biopharmaceutics review(s). Available online: https://www.accessdata.fda.gov/drugsatfda_docs/nda/2012/203415Orig1s000ClinPharmR.pdf (accessed on 24 September 2021).
22. Lankheet, N.A.G.; Knapen, L.M.; Schellens, J.H.M.; Beijnen, J.H.; Steeghs, N.; Huitema, A.D.R. Plasma concentrations of tyrosine kinase inhibitors imatinib, erlotinib, and sunitinib in routine clinical outpatient cancer care. *Ther. Drug. Monit.* **2014**, *36*, 326-334, https://doi.org/10.1097/ftd.0000000000000004.
23. Horak, J.; White, J.; Harris, A.L.; Verrill, M.; Carmichael, J.; Holt, A.; Cantarini, M.; Macpherson, M.; Swaisland, A.; Swaisland, H.; et al. The effect of different etiologies of hepatic impairment on the pharmacokinetics of gefitinib. *Cancer Chemother. Pharmacol.* **2011**, *68*, 1485-1495, https://doi.org/10.1007/s00280-011-1611-2.
24. Food and Drug Administration. Center for drug evaluation and research. Ibrutinib. Clinical pharmacology and biopharmaceutics review(s). Available online: https://www.accessdata.fda.gov/drugsatfda_docs/nda/2014/205552Orig2s000ClinPharmR.pdf (accessed on 24 September 2021).
25. Ramanathan, S.; Jin, F.; Sharma, S.; Kearney, B.P. Clinical pharmacokinetic and pharmacodynamic profile of idelalisib. *Clin. Pharmacokinet.* **2015**, *55*, 33-45, https://doi.org/10.1007/s40262-015-0304-0.
26. Farag, S.; Verheijen, R.B.; Martijn Kerst, J.; Cats, A.; Huitema, A.D.; Steeghs, N. Imatinib pharmacokinetics in a large observational cohort of gastrointestinal stromal tumour patients. *Clin. Pharmacokinet.* **2017**, *56*, 287-292, https://doi.org/10.1007/s40262-016-0439-7.
27. Yu, H.; Steeghs, N.; Nijenhuis, C.M.; Schellens, J.H.; Beijnen, J.H.; Huitema, A.D. Practical guidelines for therapeutic drug monitoring of anticancer tyrosine kinase inhibitors: focus on the pharmacokinetic targets. *Clin. Pharmacokinet.* **2014**, *53*, 305-325, https://doi.org/10.1007/s40262-014-0137-2.
28. Blum, W.; Klisovic, R.B.; Becker, H.; Yang, X.; Rozewski, D.M.; Phelps, M.A.; Garzon, R.; Walker, A.; Chandler, J.C.; Whitman, S.P.; et al. Dose escalation of lenalidomide in relapsed or refractory acute leukemias. *J. Clin. Oncol.* **2010**, *28*, 4919-4925, https://doi.org/10.1200/JCO.2010.30.3339.
29. European medicines agency. Lenvatinib. Assessment report. Available online: https://www.ema.europa.eu/en/documents/assessment-report/lenvima-epar-public-assessment-report_en.pdf (accessed on 24 September 2021).
30. Food and Drug Administration. Center for drug evaluation and research. Lorlatinib. Multi-discipline review. Available online: https://www.accessdata.fda.gov/drugsatfda_docs/nda/2018/210868Orig1s000MultidisciplineR.pdf (accessed on 24 September 2021).
31. European medicines agency. Midostaurin. Assessment report. Available online: https://www.ema.europa.eu/en/documents/assessment-report/rydapt-epar-public-assessment-report_en.pdf (accessed on 24 September 2021).
32. Food and Drug Administration. Center for drug evaluation and research. Neratinib. Multi-discipline review. Available online: https://www.accessdata.fda.gov/drugsatfda_docs/nda/2017/208051Orig1s000MultidisciplineR.pdf (accessed on 24 September 2021).
33. Giles, F.J.; Yin, O.Q.; Sallas, W.M.; le Coutre, P.D.; Woodman, R.C.; Ottmann, O.G.; Baccarani, M.; Kantarjian, H.M. Nilotinib population pharmacokinetics and exposure-response analysis in patients with imatinib-resistant or -intolerant chronic myeloid leukemia. *Eur. J. Clin. Pharmacol.* **2013**, *69*, 813-823, https://doi.org/10.1007/s00228-012-1385-4.
34. Food and Drug Administration. Center for drug evaluation and research. Nintedanib. Clinical pharmacology and biopharmaceutics review(s) Available online: https://www.accessdata.fda.gov/drugsatfda_docs/nda/2014/205832Orig1s000ClinPharmR.pdf (accessed on 24 September 2021).
35. Zhou, D.; Li, J.; Bui, K.; Learoyd, M.; Berges, A.; Milenkova, T.; Al-Huniti, N.; Tomkinson, H.; Xu, H. Bridging olaparib capsule and tablet formulations using population pharmacokinetic meta-analysis in oncology patients. *Clin. Pharmacokinet.* **2019**, *58*, 615-625, https://doi.org/10.1007/s40262-018-0714-x.
36. Food and Drug Administration. Center for drug evaluation and research. Osimertinib. Clinical pharmacology and biopharmaceutics review(s) Available online: https://www.accessdata.fda.gov/drugsatfda_docs/nda/2015/208065Orig1s000ClinPharmR.pdf (accessed on 24 September 2021).
37. Food and Drug Administration. Center for drug evaluation and research. Palbociclib. Clinical pharmacology and biopharmaceutics review(s) Available online: https://www.accessdata.fda.gov/drugsatfda_docs/nda/2015/207103Orig1s000ClinPharmR.pdf (accessed on 24 September 2021).
38. Shao, W.; Growney, J.D.; Feng, Y.; O'Connor, G.; Pu, M.; Zhu, W.; Yao, Y.-M.; Kwon, P.; Fawell, S.; Atadja, P. Activity of deacetylase inhibitor panobinostat (LBH589) in cutaneous T-cell lymphoma models: defining molecular mechanisms of resistance. *Int. J. Cancer* **2010**, *127*, 2199-2208, https://doi.org/10.1002/ijc.25218.
39. Verheijen, R.B.; Bins, S.; Mathijssen, R.H.; Lolkema, M.P.; van Doorn, L.; Schellens, J.H.; Beijnen, J.H.; Langenberg, M.H.; Huitema, A.D.; Steeghs, N.; et al. Individualized pazopanib dosing: a prospective feasibility study in cancer patients. *Clin. Cancer Res.* **2016**, *22*, 5738-5746, https://doi.org/10.1158/1078-0432.CCR-16-1255.
40. Li, Y.; Kassir, N.; Wang, X.; Palmisano, M.; Zhou, S. Population pharmacokinetics and exposure response analysis of pomalidomide in subjects with relapsed or refractory multiple myeloma from the novel combination treatment of pomalidomide, bortezomib, and low-dose dexamethasone. *J. Clin. Pharmacol.* **2020**, *60*, 1061-1075, https://doi.org/10.1002/jcph.1602.

41. Food and Drug Administration. Center for drug evaluation and research. Ponatinib. Clinical pharmacology and biopharmaceutics review(s) Available online: https://www.accessdata.fda.gov/drugsatfda_docs/nda/2012/203469Orig1s000ClinPharmR.pdf (accessed on 24 September 2021).
42. Food and Drug Administration. Center for drug evaluation and research. Regorafenib. Clinical pharmacology and biopharmaceutics review(s) Available online: https://www.accessdata.fda.gov/drugsatfda_docs/nda/2012/203085Orig1s000ClinPharmR.pdf (accessed on 24 September 2021).
43. Food and Drug Administration. Center for drug evaluation and research. Ribociclib. Multi-discipline review. Available online: https://www.accessdata.fda.gov/drugsatfda_docs/nda/2017/209092Orig1s000MultidisciplineR.pdf (accessed on 24 September 2021).
44. Food and Drug Administration. Center for drug evaluation and research. Rucaparib. Multi-discipline review. Available online: https://www.accessdata.fda.gov/drugsatfda_docs/nda/2016/209115Orig1s000MultiDisciplineR.pdf (accessed on 24 September 2021).
45. Food and Drug Administration. Center for drug evaluation and research. Ruxolitinib. Clinical pharmacology and biopharmaceutics review(s) Available online: https://www.accessdata.fda.gov/drugsatfda_docs/nda/2011/202192Orig1s000ClinPharmR.pdf (accessed on 24 September 2021).
46. Food and Drug Administration. Center for drug evaluation and research. Sonidegib. Clinical pharmacology and biopharmaceutics review(s) Available online: https://www.accessdata.fda.gov/drugsatfda_docs/nda/2015/205266Orig1s000ClinPharmR.pdf (accessed on 24 September 2021).
47. Minami, H.; Kawada, K.; Ebi, H.; Kitagawa, K.; Kim, Y.-I.; Araki, K.; Mukai, H.; Tahara, M.; Nakajima, H.; Nakajima, K. Phase I and pharmacokinetic study of sorafenib, an oral multikinase inhibitor, in japanese patients with advanced refractory solid tumors. *Cancer Sci.* **2008**, *99*, 1492-1498, https://doi.org/10.1111/j.1349-7006.2008.00837.x.
48. Teo, S.K.; Colburn, W.A.; Tracewell, W.G.; Kook, K.A.; Stirling, D.I.; Jaworsky, M.S.; Scheffler, M.A.; Thomas, S.D.; Laskin, O.L. Clinical pharmacokinetics of thalidomide. *Clin. Pharmacokinet.* **2004**, *43*, 311-327, https://doi.org/10.2165/00003088-200443050-00004.
49. Fishman, M.N.; Srinivas, S.; Hauke, R.J.; Amato, R.J.; Esteves, B.; Cotreau, M.M.; Strahs, A.L.; Slichenmyer, W.J.; Bhargava, P.; Kabbinavar, F.F. Phase Ib study of tivozanib (AV-951) in combination with temsirolimus in patients with renal cell carcinoma. *Eur. J. Cancer* **2013**, *49*, 2841-2850, https://doi.org/10.1016/j.ejca.2013.04.019.
50. Food and Drug Administration. Center for drug evaluation and research. Trametinib. Clinical pharmacology and biopharmaceutics review(s) Available online: https://www.accessdata.fda.gov/drugsatfda_docs/nda/2013/204114Orig1s000ClinPharmR.pdf (accessed on 24 September 2021).
51. Food and Drug Administration. Center for drug evaluation and research. Trifluridine. Pharmacology review(s). Available online: https://www.accessdata.fda.gov/drugsatfda_docs/nda/2015/207981Orig1s000PharmR.pdf (accessed on 24 September 2021).
52. Food and Drug Administration. Center for drug evaluation and research. Vandetanib. Clinical pharmacology and biopharmaceutics review(s) Available online: https://www.accessdata.fda.gov/drugsatfda_docs/nda/2011/022405Orig1s000ClinPharmR.pdf (accessed on 24 September 2021).
53. Food and Drug Administration. Center for drug evaluation and research. Vemurafenib. Clinical pharmacology and biopharmaceutics review(s) Available online: https://www.accessdata.fda.gov/drugsatfda_docs/nda/2011/202429Orig1s000ClinPharmR.pdf (accessed on 24 September 2021).
54. Reda, G.; Cassin, R.; Dovrtelova, G.; Matteo, C.; Giannotta, J.; D'Incalci, M.; Cortelezzi, A.; Zucchetti, M. Venetoclax penetrates in cerebrospinal fluid and may be effective in chronic lymphocytic leukemia with central nervous system involvement. *Haematologica* **2019**, *104*, e222-e223, https://doi.org/10.3324/haematol.2018.213157.
55. Corona, G.; Gusella, M.; Gaspardo, A.; Miolo, G.; Bertolaso, L.; Pezzolo, E.; Pasini, F.; Steffan, A.; Caruso, D. Rapid LC-MS/MS method for quantification of vinorelbine and 4-O-deacetylvinorelbine in human whole blood suitable to monitoring oral metronomic anticancer therapy. *Biomed. Chromatogr.* **2018**, e4282, https://doi.org/10.1002/bmc.4282.
56. U.S. Food and Drug Administration (FDA). Bioanalytical method validation guidance for industry. 2018. Available online: https://www.fda.gov/files/drugs/published/Bioanalytical-Method-Validation-Guidance-for-Industry.pdf (accessed on 24 September 2021).
